# Numerical Determination of the Secondary Acoustic Radiation Force on a Small Sphere in a Plane Standing Wave Field

**DOI:** 10.3390/mi10070431

**Published:** 2019-06-29

**Authors:** Gergely Simon, Marco A. B. Andrade, Marc P. Y. Desmulliez, Mathis O. Riehle, Anne L. Bernassau

**Affiliations:** 1 School of Engineering and Physical Sciences, Heriot-Watt University, Edinburgh EH14 4AS, UK; 2 OnScale Ltd., Glasgow G2 5QR, UK; 3 Institute of Physics, University of São Paulo, São Paulo 05508-090, Brazil; 4 Institute of Molecular Cell and Systems Biology, Centre for Cell Engineering, University of Glasgow, Glasgow G12 8QQ, UK

**Keywords:** secondary acoustic radiation force, scattering effects, 2D and 3D modeling

## Abstract

Two numerical methods based on the Finite Element Method are presented for calculating the secondary acoustic radiation force between interacting spherical particles. The first model only considers the acoustic waves scattering off a single particle, while the second model includes re-scattering effects between the two interacting spheres. The 2D axisymmetric simplified model combines the Gor’kov potential approach with acoustic simulations to find the interacting forces between two small compressible spheres in an inviscid fluid. The second model is based on 3D simulations of the acoustic field and uses the tensor integral method for direct calculation of the force. The results obtained by both models are compared with analytical equations, showing good agreement between them. The 2D and 3D models take, respectively, seconds and tens of seconds to achieve a convergence error of less than 1%. In comparison with previous models, the numerical methods presented herein can be easily implemented in commercial Finite Element software packages, where surface integrals are available, making it a suitable tool for investigating interparticle forces in acoustic manipulation devices.

## 1. Introduction

Acoustic radiation forces generated by standing wave fields are widely used for manipulation, trapping, patterning, and sorting of microparticles and cells [1,2,3,4]. The theoretical investigation of the primary acoustic radiation force on microparticles due to an external acoustic field was introduced by King [5], who calculated the acoustic radiation force on a small rigid sphere in an ideal fluid for both traveling and standing plane wave fields. His work was followed by the paper of Yosioka and Kawasima [6], which considered the compressibility of the spheres. Gor’kov provided a potential approach for calculation of this force in an arbitrary acoustic field [7]. Recent investigations included viscous and thermal effects [8,9,10] and provided analytical formulae for more complex external fields [11,12,13].

Considering multiple particles in a fluid medium, the scattering events between these particles give rise to interparticle forces (also called secondary radiation force or Bjerknes force), which can result in particle clump formation, adversely affecting device performance [14]. Desired arrangement of particles in layers [15] or chains [16] is also suspected to occur due to the secondary radiation force [17]. Special cases of this force were investigated thoroughly: the seminal work by Bjerknes [18] on bubble-bubble interactions was followed by other theoretical studies [19,20,21,22] and validated experimentally [23]. Theoretical rigid-rigid particle interactions were developed both for short range [16] and long range interactions [24], and validated by experiments with elastic latex solid particles in water medium [25]. A general theoretical model both for compressible and rigid particles, with no restriction on interparticle distance, was presented by Silva and Bruus recently [26]. They followed a monopole-dipole description of the secondary force potential; this analytical formula being valid for particle sizes much smaller than the wavelength. To alleviate this restriction, numerical approaches have been developed for determining interparticle forces. Doinikov used a multipole series expansion technique for calculating the interaction force between two air bubbles in water [27]. In 2015, a weighted residue method was combined with the multipole expansion series for calculating the interparticle forces between spherical particles in an ideal fluid [28]. Recently, a boundary element method was applied for calculating the interparticle force between spheroidal particles [29]. Although different numerical methods have been developed, they are complex to use and require a laborious implementation, restricting their use to a few research groups. Analytical methods, in contrast, are limited to small particle sizes and objects of simple geometry.

In this paper, we present two numerical approaches to calculate the secondary acoustic radiation force on small spheres in a plane standing wave field. One uses a 2D axisymmetric model and the Gor’kov potential approach for calculation of the force [7], while the second model uses a tensor integral approach [6,30] in 3D and therefore can include re-scattering events between the particles as well. Both approaches use the Finite Element Method (FEM) to calculate the acoustic pressure and particle velocity distributions in the fluid medium. The obtained numerical results are compared with those obtained by an analytical expression for the secondary force acting on particles much smaller than the acoustic wavelength [26]. The 2D axisymmetric model has the advantage of being simple to implement while the 3D model can be extended for particles with arbitrary shapes and sizes.

## 2. Methods

This section presents the analytical expressions of the primary and secondary radiation forces on small spheres in an ideal fluid medium. Assuming time-harmonic fields of angular frequency ω, the wave propagation can be described in terms of the velocity potential $\phi \left(r,t\right)=\phi \left(r\right)e^{-i\omega t}$ [26], where r is the position vector, t is the time and $i=\sqrt{-1}$ is the imaginary unit. Given ϕ(r,t), the acoustic pressure p(r,t) and velocity v(r,t) fields can be fully specified using a complex amplitude [31]:

(1a) $$v\left(r,t\right)=\nabla \phi \left(r,t\right)=\nabla \phi \left(r\right)e^{-i\omega t}=v\left(r\right)e^{-i\omega t},$$

(1b) v(r)=∇ϕ(r),

(2a) $$p\left(r,t\right)=-\rho_{0}\frac{\partial \phi \left(r,t\right)}{\partial t}=-\rho_{0}\phi \left(r\right)\frac{\partial e^{-i\omega t}}{\partial t}=\rho_{0}\phi \left(r\right)i\omega e^{-i\omega t}=p\left(r\right)e^{-i\omega t},$$

(2b) $$p\left(r\right)=i\omega \rho_{0}\phi \left(r\right),$$

where $\rho_{0}$ is the density of the unperturbed fluid.

An expression for calculating the acoustic radiation force produced by an arbitrary acoustic field (except a plane traveling wave) on a small sphere was derived by Gor’kov [7]. According to Gor’kov’s potential theory, the acoustic radiation force $F_{\mathrm{rad}}$ on a small spherical object of radius a, below the Rayleigh scattering limit (a≪λ), can be expressed in the form of a time-averaged potential [32], given by

(3a) $$U_{\mathrm{rad}}\left(r\right)=V_{i}\left[\frac{1}{2}f_{0,i}\kappa_{0}\langle {\left|p_{\mathrm{in}}\left(r,t\right)\right|}^{2}\rangle -\frac{3}{4}f_{1,i}\rho_{0}\langle {\left|v_{\mathrm{in}}\left(r,t\right)\right|}^{2}\rangle \right],$$

(3b) $$F_{\mathrm{rad}}\left(r\right)=-\nabla U_{\mathrm{rad}}\left(r\right),$$

where $p_{\mathrm{in}}$ and $v_{\mathrm{in}}$ are the incident pressure and velocity fields at the position where the spherical object is located. In Equation (3a), $V_{i}=4\pi a_{i}^{3}/3$ is the particle volume, $\kappa_{0}=1/\left(\rho_{0}c_{0}^{2}\right)$ is the compressibility of the fluid medium, 〈〉 denotes time averaging for one period, and

(4a) $$f_{0,i}=1-{\tilde{\kappa }}_{i}\;\mathrm{with}\;{\tilde{\kappa }}_{i}=\kappa_{i}/\kappa_{0},$$

(4b) $$f_{1,i}=\frac{2\left({\tilde{\rho }}_{i}-1\right)}{2{\tilde{\rho }}_{i}+1}\;\mathrm{with}\;{\tilde{\rho }}_{i}=\rho_{i}/\rho_{0},$$

are the monopole and dipole scattering factors, respectively, with $\kappa_{i}$ being the compressibility, $\rho_{i}$ the density of the particle [32].

### 2.1. Theoretical Expression for the Primary Acoustic Radiation Force

**Figure 1:**
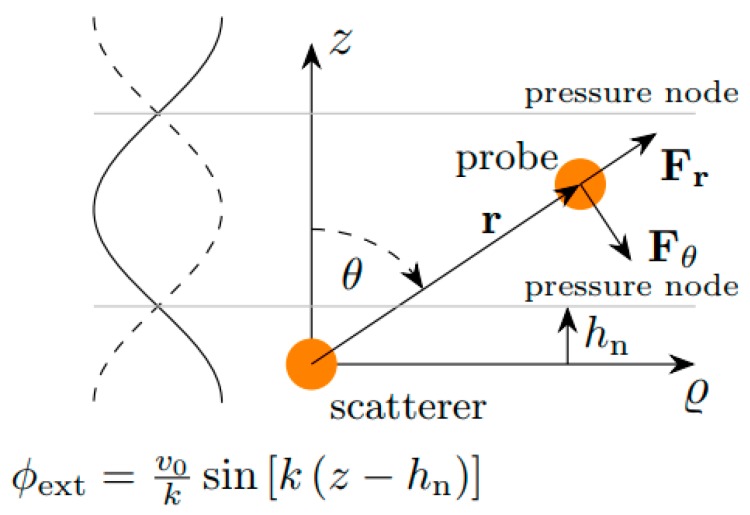
Two small particles in a plane standing wave field. The total acoustic radiation force on the probe particle is the result of a primary radiation force and a secondary force. The primary force originates from the interaction of the external wave field with the probe particle, while the secondary force is generated by the scattered wave from the scatterer particle.

Let us assume a small probe particle in an external standing plane wave field, as illustrated in Figure 1. The velocity potential $\phi_{\mathrm{ext}}$ of a plane standing wave field at the location of the probe particle can be described by [26]:

(5) $$\phi_{\mathrm{ext}}\left(z\right)=\frac{v_{0}}{k}\sin\left[k\left(z-h_{n}\right)\right],$$

where k=2π/λ is the wavenumber, $v_{0}$ is the velocity amplitude, and $h_{n}$ is the distance between the scatterer particle and the pressure node. Substituting the velocity potential given by Equation (5) into Equations (1b) and (2b), we find the particle velocity $v_{\mathrm{ext}}\left(z\right)$ and the acoustic pressure $p_{\mathrm{ext}}\left(z\right)$:

(6) $$v_{\mathrm{ext}}\left(z\right)=\nabla \phi_{\mathrm{ext}}\left(z\right)=v_{0}\cos\left[k\left(z-h_{n}\right)\right]\hat{z},$$

(7) $$p_{\mathrm{ext}}\left(z\right)=i\omega \rho_{0}\phi_{\mathrm{ext}}\left(z\right)=i\omega \rho_{0}\frac{v_{0}}{k}\sin\left[k\left(z-h_{n}\right)\right],$$

Substituting Equations (6) and (7) into Equation (3a), and as the time averages of the squares of the fields are half of the amplitude squared, moreover $\omega =kc_{0}$ the potential of the primary radiation force is

(8) $$U_{\mathrm{prim}}\left(r\right)=\frac{V_{i}E_{0}}{2}\left[f_{0,i}-\Phi_{\mathrm{AC}}\cos^{2}\left[k\left(z-h_{n}\right)\right]\right],$$

where $E_{0}=\frac{1}{2}\rho_{0}v_{0}^{2}=\frac{1}{2}\kappa_{0}p_{0}^{2}$ is the acoustic energy density and $\Phi_{\mathrm{AC}}$ is the acoustic contrast factor, given by:

(9) $$\Phi_{\mathrm{AC}}=f_{0,i}+\frac{3}{2}f_{1,i}=\frac{5{\tilde{\rho }}_{i}-2}{2{\tilde{\rho }}_{i}+1}-{\tilde{\kappa }}_{i}.$$

Finally, the primary acoustic radiation force acting on the probe particle is obtained by substituting Equation (8) in Equation (3b):

(10) $$F_{\mathrm{prim}}=-\nabla U_{\mathrm{ext}}\left(r\right)=-\frac{V_{i}E_{0}\Phi_{\mathrm{AC}}k}{2}\sin\left[2k\left(z-h_{n}\right)\right]\hat{z}.$$

The primary radiation force points towards the pressure node if the contrast factor is positive and points towards the antinode if the contrast factor is negative.

### 2.2. Theoretical Expression for the Potential of the Secondary Force

In addition to the primary force, a particle located in the neighborhood of another particle experiences a secondary force, which is caused by the wave scattering from the neighboring particle. A general expression for the secondary force potential $U_{\sec}$ acting on the probe particle due to a scatterer particle located at the origin of the coordinate system (See Figure 1) was derived by Silva and Bruus [26]:

(11) $$U_{\sec}(r,\theta )=\pi E_{0}k^{3}a_{p}^{3}a_{s}^{3}(\cos\left[k\left(r\cos\theta -h_{n}\right)]\frac{f_{1,p}}{2}\{f_{1,s}\cos\left(kh_{n}\right)\left(1+3\cos2\theta \right)\frac{\cos kr}{{\left(kr\right)}^{3}}\;+\left[\frac{4}{3}f_{0,s}\sin\left(kh_{n}\right)\cos\theta \cos kr+f_{1,s}\cos\left(kh_{n}\right)\left(1+3\cos2\theta \right)\sin kr\right]\frac{1}{{\left(kr\right)}^{2}}\;-\left[f_{1,s}\cos\left(kh_{n}\right)\left(1+\cos2\theta \right)\cos kr-\frac{4}{3}f_{0,s}\sin\left(kh_{n}\right)\cos\theta \sin kr\right]\frac{1}{kr}\}\;+\sin\left[k\left(r\cos\theta -h_{n}\right)\right]\frac{2f_{0,p}}{3}\{f_{1,s}\cos\left(kh_{n}\right)\cos\theta \frac{\cos kr}{{\left(kr\right)}^{2}}\;+\left[\frac{2}{3}f_{0,s}\sin\left(kh_{n}\right)\cos kr+f_{1,s}\cos\left(kh_{n}\right)\cos\theta \sin kr\right]\frac{1}{kr}\}\right)$$

where $a_{p}$ and $a_{s}$ are the radius of the probe and scatterer particles, respectively. This potential can be inserted into Equation (3b) to obtain the total secondary radiation force (refer to Figure 1 for the components of the force):

(12) $$F_{r}=\pi E_{0}k^{3}a_{p}^{3}a_{s}^{3}\{\frac{f_{1,p}}{2}\cos[k\left(r\cos\theta -h_{n}\right)]\{f_{1,s}\cos kh_{n}\left(1+3\cos2\theta \right)\;\left[\frac{3k\cos kr}{{\left(kr\right)}^{4}}+\frac{3k\sin kr}{{\left(kr\right)}^{3}}-\frac{k\cos kr}{{\left(kr\right)}^{2}}\right]+\frac{4}{3}f_{0,s}\sin kh_{n}\cos\theta [\frac{2k\cos kr}{{\left(kr\right)}^{3}}+\frac{2k\sin kr}{{\left(kr\right)}^{2}}-\frac{k\cos kr}{kr}]\;-f_{1,s}\cos kh_{n}\left(1+\cos2\theta \right)[\frac{k\cos kr}{{\left(kr\right)}^{2}}+\frac{k\sin kr}{kr}]\}+\frac{2f_{0,p}}{3}\cos[k\left(r\cos\theta -h_{n}\right)]\;\{f_{1,s}\cos kh_{n}\cos\theta \left[-\frac{k\cos\theta \cos kr}{{\left(kr\right)}^{2}}-\frac{k\cos\theta \sin kr}{kr}\right]+\frac{2}{3}f_{0,s}\sin kh_{n}\left[-\frac{k\cos\theta \cos kr}{kr}\right]\}+\;\frac{f_{1,p}}{2}\sin[k\left(r\cos\theta -h_{n}\right)]\{f_{1,s}\cos kh_{n}\left(1+3\cos2\theta \right)[\frac{k\cos\theta \cos kr}{{\left(kr\right)}^{3}}+\frac{k\cos\theta \sin kr}{{\left(kr\right)}^{2}}]+\;\frac{4}{3}f_{0,s}\sin kh_{n}\cos\theta [\frac{k\cos\theta \cos kr}{{\left(kr\right)}^{2}}+\frac{k\cos\theta \sin kr}{kr}]-f_{1,s}\cos kh_{n}\left(1+\cos2\theta \right)[\frac{k\cos\theta \cos kr}{kr}]\}+\;\frac{2f_{0,p}}{3}\sin\left[k\left(r\cos\theta -h_{n}\right)\right]\{f_{1,s}\cos kh_{n}\cos\theta [\frac{2k\cos kr}{{\left(kr\right)}^{3}}+\frac{2k\sin kr}{{\left(kr\right)}^{2}}-\frac{k\cos kr}{kr}]+\frac{2}{3}f_{0,s}\sin kh_{n}[\frac{k\cos kr}{{\left(kr\right)}^{2}}+\frac{k\sin kr}{kr}]\}\}\hat{r}$$

(13) $$F_{\theta }=\frac{1}{r}\pi E_{0}k^{3}a_{p}^{3}a_{s}^{3}\{\frac{f_{1,p}}{2}\cos[k\left(r\cos\theta -h_{n}\right)]\{f_{1,s}\cos kh_{n}\frac{\cos kr}{{\left(kr\right)}^{3}}\left[6\sin2\theta \right]+\;\frac{4}{3}f_{0,s}\sin kh_{n}\frac{\cos kr}{{\left(kr\right)}^{2}}[\sin\theta ]+f_{1,s}\cos kh_{n}\frac{\sin kr}{{\left(kr\right)}^{2}}\left[6\sin2\theta \right]-f_{1,s}\cos kh_{n}\frac{\cos kr}{kr}[2\sin2\theta ]+\;\frac{4}{3}f_{0,s}\sin kh_{n}\frac{\sin kr}{kr}\left[\sin\theta \right]\}+\frac{2f_{0,p}}{3}\cos\left[k\left(r\cos\theta -h_{n}\right)\right]\{f_{1,s}\cos kh_{n}\frac{\cos kr}{{\left(kr\right)}^{2}}\left[kr\sin\theta \cos\theta \right]+\;\frac{2}{3}f_{0,s}\sin kh_{n}\frac{\cos kr}{kr}\left[kr\sin\theta \right]+f_{1,s}\cos kh_{n}\frac{\sin kr}{kr}\left[kr\sin\theta \cos\theta \right]+\frac{f_{1,p}}{2}\sin\left[k\left(r\cos\theta -h_{n}\right)\right]\;\{f_{1,s}\cos kh_{n}\frac{\cos kr}{{\left(kr\right)}^{3}}\left[-kr\sin\theta \left(1+3\cos2\theta \right)\right]+\frac{4}{3}f_{0,s}\sin kh_{n}\frac{\cos kr}{{\left(kr\right)}^{2}}\left[-kr\sin\theta \cos\theta \right]+\;f_{1,s}\cos kh_{n}\frac{\sin kr}{{\left(kr\right)}^{2}}\left[-kr\sin\theta \left(1+3\cos2\theta \right)\right]-f_{1,s}\cos kh_{n}\frac{\cos kr}{kr}\left[-kr\sin\theta \left(1+\cos2\theta \right)\right]\;+\frac{4}{3}f_{0,s}\sin kh_{n}\frac{\sin kr}{kr}\left[-kr\sin\theta \cos\theta ]\right\}+\frac{2f_{0,p}}{3}\sin\left[k\left(r\cos\theta -h_{n}\right)\right]\;\left\{f_{1,s}\cos kh_{n}\frac{\cos kr}{{\left(kr\right)}^{2}}\left[\sin\theta \right]+f_{1,s}\cos kh_{n}\frac{\sin kr}{kr}\left[\sin\theta \right]\right\}\hat{\theta }.$$

The radial force for the transversal direction (θ=π/2) simplifies to

(14) $$F\left(\varrho \right)=\pi E_{0}k^{3}a_{p}^{3}a_{s}^{3}[f_{1,p}f_{1,s}\cos^{2}\left(kh_{n}\right)\left(-\frac{3k\cos k\varrho }{{\left(k\varrho \right)}^{4}}-\frac{3k\sin k\varrho }{{\left(k\varrho \right)}^{3}}+\frac{k\cos k\varrho }{{\left(k\varrho \right)}^{2}}\right)+\;\frac{4}{9}f_{0,p}f_{0,s}\sin^{2}\left(kh_{n}\right)\left(-\frac{k\cos k\varrho }{{\left(k\varrho \right)}^{2}}-\frac{k\sin k\varrho }{k\varrho }\right)]\hat{\varrho },$$

where the first term corresponds to the dipole scattering and the second term corresponds to the monopole scattering. Choosing the nodal line to align with the transverse plane, the monopole scattering term vanishes, and for the antinodal line the dipole term vanishes. Therefore, in the short and long range the force is

(15a) $$F\left(\varrho \right)=-\pi E_{0}k^{3}a_{p}^{3}a_{s}^{3}f_{1,p}f_{1,s}\frac{3k}{{\left(k\varrho \right)}^{4}}\hat{\varrho }\;\mathrm{with}\;k\varrho \ll 1,$$

(15b) $$F\left(\varrho \right)=\pi E_{0}k^{3}a_{p}^{3}a_{s}^{3}f_{1,p}f_{1,s}\frac{k\cos k\varrho }{{\left(k\varrho \right)}^{2}}\hat{\varrho }\;\mathrm{with}\;k\varrho \gg 1,$$

for the nodal plane.

For the antinodal plane the force in the two ranges is:

(16a) $$F\left(\varrho \right)=-\frac{4}{9}\pi E_{0}k^{3}a_{p}^{3}a_{s}^{3}f_{0,p}f_{0,s}\frac{k}{{\left(k\varrho \right)}^{2}}\hat{\varrho }\;\mathrm{with}\;k\varrho \ll 1,$$

(16b) $$F\left(\varrho \right)=-\frac{4}{9}\pi E_{0}k^{3}a_{p}^{3}a_{s}^{3}f_{0,p}f_{0,s}\frac{k\sin k\varrho }{k\varrho }\hat{\varrho }\;\mathrm{with}\;k\varrho \gg 1.$$

The radial force in the *z* direction (θ=0) becomes somewhat more complicated for a general case:

(17) $$F\left(z\right)=\pi E_{0}k^{3}a_{p}^{3}a_{s}^{3}\{\frac{f_{1,p}}{2}\cos\left[k\left(z-h_{n}\right)\right]\{4f_{1,s}\cos kh_{n}\left[\frac{3k\cos kz}{{\left(kz\right)}^{4}}+\frac{3k\sin kz}{{\left(kz\right)}^{3}}-\frac{k\cos kz}{{\left(kz\right)}^{2}}\right]+\;\frac{4}{3}f_{0,s}\sin kh_{n}\left[\frac{2k\cos kz}{{\left(kz\right)}^{3}}+\frac{2k\sin kz}{{\left(kz\right)}^{2}}-\frac{k\cos kz}{kz}\right]-2f_{1,s}\cos kh_{n}\left[\frac{k\cos kz}{{\left(kz\right)}^{2}}+\frac{k\sin kz}{kz}\right]\}+\;\frac{2f_{0,p}}{3}\cos\left[k\left(z-h_{n}\right)\right]\left\{f_{1,s}\cos kh_{n}\left[-\frac{k\cos kz}{{\left(kz\right)}^{2}}-\frac{k\sin kz}{kz}\right]+\frac{2}{3}f_{0,s}\sin kh_{n}\left[-\frac{k\cos kz}{kz}\right]\right\}\;+\frac{f_{1,p}}{2}\sin\left[k\left(z-h_{n}\right)\right]\left\{4f_{1,s}\cos kh_{n}\left[\frac{k\cos kz}{{\left(kz\right)}^{3}}+\frac{k\sin kz}{{\left(kz\right)}^{2}}\right]+\frac{4}{3}f_{0,s}\sin kh_{n}\left[\frac{k\cos kz}{{\left(kz\right)}^{2}}+\frac{k\sin kz}{kz}\right]-2f_{1,s}\cos kh_{n}\left[\frac{k\cos kz}{kz}\right]\right\}\;+\frac{2f_{0,p}}{3}\sin\left[k\left(z-h_{n}\right)\right]\left\{f_{1,s}\cos kh_{n}\left[\frac{2k\cos kz}{{\left(kz\right)}^{3}}+\frac{2k\sin kz}{{\left(kz\right)}^{2}}-\frac{k\cos kz}{kz}\right]+\frac{2}{3}f_{0,s}\sin kh_{n}\left[\frac{k\cos kz}{{\left(kz\right)}^{2}}+\frac{k\sin kz}{kz}\right]\right\}\}\hat{z}$$

which simplifies to

(18) $$F\left(z\right)=\pi E_{0}k^{4}a_{p}^{3}a_{s}^{3}f_{1,s}\left\{f_{1,p}\cos kz\left[\frac{6\cos kz}{{\left(kz\right)}^{4}}+\frac{6\sin kz}{{\left(kz\right)}^{3}}-\frac{3\cos kz}{{\left(kz\right)}^{2}}-\frac{\sin kz}{kz}\right]-\;\frac{2f_{0,p}}{3}\cos kz\left[\frac{\cos kz}{{\left(kz\right)}^{2}}+\frac{\sin kz}{kz}\right]+\left(f_{1,p}+\frac{2f_{0,p}}{3}\right)\sin kz\left[\frac{2\cos kz}{{\left(kz\right)}^{3}}+\frac{2\sin kz}{{\left(kz\right)}^{2}}-\frac{\cos kz}{kz}\right]\right\}\hat{z},$$

when the nodes are aligned with the scatterer particle ($h_{n}=0$). Please note that this force is directly proportional to the dipole scattering coefficient of the scatterer particle.

When the scatterer sphere is located at the pressure antinode ($h_{n}=\lambda /4$), the secondary force in the *z* direction is

(19) $$F\left(z\right)=\pi E_{0}k^{4}a_{p}^{3}a_{s}^{3}f_{0,s}\left\{\frac{2}{3}f_{1,p}\sin kz\left[\frac{2\cos kz}{{\left(kz\right)}^{3}}+\frac{2\sin kz}{{\left(kz\right)}^{2}}\right]\;-\left(\frac{2}{3}f_{1,p}+\frac{4f_{0,p}}{9}\right)\sin kz\left[\frac{\cos kz}{kz}\right]-\left(\frac{2}{3}f_{1,p}+\frac{4f_{0,p}}{9}\right)\cos kz\left[\frac{\cos kz}{{\left(kz\right)}^{2}}+\frac{\sin kz}{kz}\right]\right\}\hat{z}.$$

Please note that this force is directly proportional to the monopole scattering coefficient of the scatterer particle.

Similar simplifications can be performed for the force in the polar direction. Please note that all terms in Equation (13b) either contain sinθ or sin2θ and therefore the force goes to zero for θ=0. However, for probe particles located along the transversal plane (θ=π/2), this force will have the form

(20) $$F_{\theta }=\pi E_{0}k^{4}a_{p}^{3}a_{s}^{3}\frac{\sin2kh_{n}}{18}\left[\left(6f_{1,p}f_{0,s}-9f_{1,p}f_{1,s}-6f_{0,p}f_{1,s}\right)\left(\frac{\cos kr}{{\left(kr\right)}^{3}}+\frac{\sin kr}{{\left(kr\right)}^{2}}\right)+4f_{0,p}f_{0,s}\frac{\cos kr}{kr}\right]\hat{\theta }$$

whose value is zero when the scatterer particle is either at a node or antinode, since $\sin2kh_{n}=0$ in these cases.

### 2.3. Determination of the Secondary Force by the Finite Element Method

The secondary acoustic radiation force acting on a small probe particle was determined by two types of FEM simulations. The first one, presented in Section 3.1, uses a 2D axisymmetric FEM model to simulate the first order acoustic pressure and velocity distribution in the neighborhood of a scatterer particle. These fields are substituted in the Gor’kov expression to find the radiation force on a small probe particle. The second strategy combines a 3D FEM model and a tensor integral approach to calculate the radiation force on the probe particle. This strategy, presented in Section 3.2, considers re-scattering events between the particles as well. Both types of simulations were implemented in the FEM software COMSOL Multiphysics (version 5.2a, COMSOL AB, Stockholm, Sweden).

### 2.4. Simplified Numerical Approach

**Figure 2:**
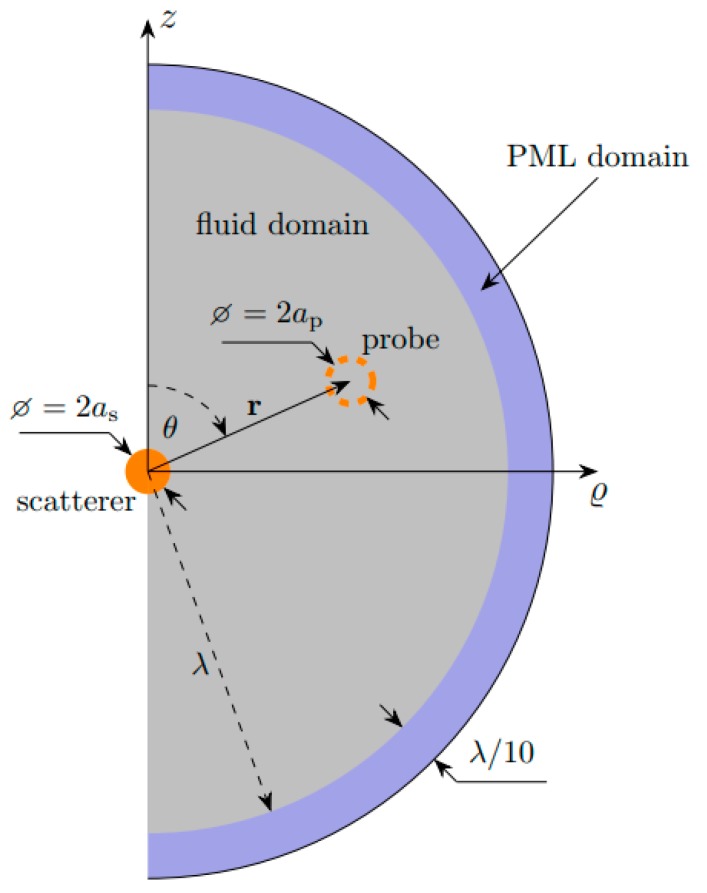
Axisymmetric linear acoustic model used to calculate the secondary force on the probe particle. The model, based on the Gor’kov expression, is used to calculate the incident acoustic pressure and velocity fields at the probe particle located at $r=z\hat{z}+\varrho \hat{\varrho }$.

The first numerical approach, based on the Gor’kov expression, is presented in Figure 2. The scatterer sphere is located at the origin of the coordinate system and we are interested in determining the secondary force on a small probe particle, located at the position $r=z\hat{z}+\varrho \hat{\varrho }$, due to an external plane standing wave field.

As the probe particle is neglected in the simulations and only the incident fields are calculated at its location, the model has rotational symmetry and was set up as a 2D axisymmetric problem.

In the acoustics module of COMSOL Multiphysics, the external incident field was introduced as a background pressure field and the model was used to calculate the acoustic pressure and the velocity distribution caused by the superposition of the external field with the scattered field from the scatterer particle. As shown in Figure 2, a perfectly matched layer (PML) was used for absorption of the acoustic waves at the edge of the fluid domain. In COMSOL, the total pressure is described by its amplitude *p*_t_, while the total velocity by its root mean square value *v*_rms_. Consequently, for the harmonic field, the total potential at the probe particle is given by

(21) $$U_{\mathrm{total}}\left(r\right)=U_{\mathrm{prim}}\left(r\right)+U_{\sec}\left(r\right)=\frac{V_{i}}{4}\left[f_{0,i}\kappa_{0}{\left|p_{t}\left(r\right)\right|}^{2}-3f_{1,i}\rho_{0}v_{\mathrm{rms}}^{2}\left(r\right)\right]$$

where *U*_prim_ is the potential due to the external field, and Usec is the potential due to the re-scattered field.

From the total potential, given by Equation (21), and the potential of the primary force, given by Equation (9), the secondary potential and force acting on the probe particle are given by

(22a) $$U_{\sec}\left(r\right)=U_{\mathrm{total}}\left(r\right)-U_{\mathrm{prim}}\left(r\right),$$

(22b) $$F_{\sec}\left(r\right)=-\nabla U_{\sec}\left(r\right).$$

The maximum mesh size was chosen to be *λ*/23, and the discretization was set to quartic to efficiently increase the degrees of the freedom and capture the problem. This resulted in around 50,000 to 100,000 degrees of freedom for the model. More details on the meshing and its convergence is provided in the Supplementary Materials. As a single simulation can be used to obtain the effect of one scatterer particle on all probe particles, a total of M simulations are required for the full characterization of the interparticle forces between M particles.

The computational requirement on a general purpose PC equipped with 8 GB RAM was between 2 to 10 s for 2000 and 200,000 Degrees of Freedom (DOFs), respectively. However, as shown in the Supplementary Materials, convergence error less than 0.1% is achieved even for 60,000 DOFs.

### 2.5. Complete Finite Element Model Incorporating Re-Scattering Effects

**Figure 3:**
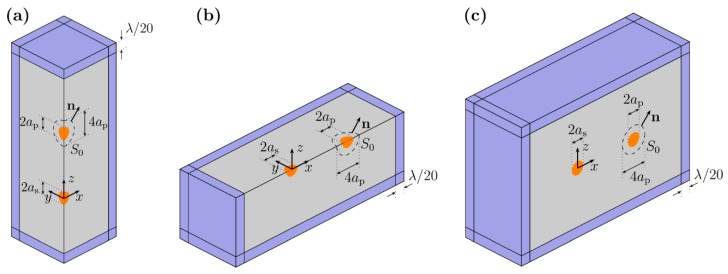
Schematics of the models used in COMSOL with the geometrical dimensions indicated. The models used in the 3D simulations, making use of different symmetries of the problem. The perfectly matched layer is indicated in blue and the main fluid domain is shaded in grey. All grey faces have symmetry boundary conditions applied (**a**) For simulations along the *z* direction, (**b**) For simulations along the radial direction, for *h*_n_ = *λ*/4, (**c**) For simulations along the radial direction, for *h*_n_ = 0.

Both the monopole-dipole-based theoretical and the previous simulation approach neglect the re-scattering effects between particles. Therefore, to assess the importance of these events, the following 3D model was implemented in COMSOL. As the FEM simulation is a direct numerical solution of the Helmholtz equation with appropriate boundary conditions at the surfaces, re-scattering effects are directly included when the solutions are obtained [33]. First, the pressure field is simulated including both the scatterer and the probe particles, followed by the evaluation of the force using the tensor integral method over a bounding surface. As in the previous case, the incident standing wave is represented by the background field given by Equation (7). The total acoustic radiation force acting on the probe particle is [30]:

(23) $$F_{\mathrm{total}}=-\overset{}{\underset{S_{0}}{\iint }}\left\{\left[\frac{p^{2}}{2\rho_{0}c_{0}^{2}}-\frac{\rho_{0}{\left|v\right|}^{2}}{2}\right]n+\rho_{0}\left(n\cdot v\right)v\right\}dS,$$

where $S_{0}$ is a closed surface surrounding the particle and n is the unit vector pointing outwards the surface $S_{0}$ (see Figure 3). The approximation is valid to the second order. Although Glynne-Jones et al. [30] use Equation (23) only to obtain the primary radiation force acting on the particle, it is valid for calculating the total acoustic radiation force for arbitrary shapes, sizes and acoustic background fields. The primary radiation force arises due to the interaction of scattered waves from the particle and the background pressure field; similarly, the secondary radiation force is a result of the interaction of the scattered waves from particles i and j [9]. Therefore, using Equation (23) for a multiparticle configuration, the total force, including secondary effects, is computed.

As with the previous model, the secondary force on the probe particle can be found by subtracting the primary force from the total force:

(24) $$F_{\sec}=F_{\mathrm{total}}-F_{\mathrm{prim}},$$

where the primary force is calculated by Equation (11). As the convergence of the secondary radiation force was slow with this approach, we also calculated the primary radiation force using the same tensor integral method, but first changing the material of the scatterer particle to the liquid. We believe, the reason behind the slow convergence is the inadequately spaced mesh on the surface of the probe particle, as opposed to the multipole scattering methods [27,28], where the quadrature is a well-spaced Gauss-Langrangian quadrature. Carrying out the integral of the total force, and then changing the material of the scatterer to the fluid, the exact same mesh is used in both cases, significantly reducing computational errors and increasing convergence speed, which is shown in the Supplementary Materials. As a result, one simulation is required to obtain the total force on all particles, followed by one simulation for each particle to obtain the primary radiation force, resulting in a total number of M+1 simulations for M particles.

To reduce the computational burden, we used the symmetry of the different cases as follows. For obtaining the secondary force along the *z*-axis, we only modeled a quarter of the domain (Figure 3b) and multiplied the integration value by 4 to account for the original problem. Similarly, along direction ϱ, we only modeled half or quarter of the domain, as shown in Figure 3b,c. For an antinode at *z* = 0, both *xy* and *xz* planes are symmetry planes (Figure 3b), while a symmetry boundary condition was applied over the *xz*-plane for a node being present at *z* = 0 (Figure 3c).

To be able to capture the numerical results with a similar precision as in the other model, the mesh size was chosen to be the same. For this model, it resulted in several degrees of freedom ranging between 300,000 and 450,000. The computational time for these models reached 60 s; however adequate convergence below 1% error was already achieved with 200,000 DOFs, which required simulation time of 30 s.

This 3D model cannot be applied to touching spheres. We believe this is due to the discontinuity of the density at the touching point between the two spheres. However, as Figure S7 of the Supplementary Materials shows, simulations down to separation distances as low as 0.001*λ* are possible.

In the results section, we investigate two cases of the secondary radiation force: particle in air and particle in water. As the range of the interaction force is significantly different for these, we decided to normalize both the secondary potential and force by their primary counterpart to allow for direct comparison of the two cases by the relative values.

The primary radiation force (given by Equation (9)) amplitude is $V_{i}E_{0}\Phi_{\mathrm{AC}}k/2$. Using this value as normalization, the relative strength of the different cases to follow can be compared with ease.

## 3. Results

To underpin the versatility of the model, we show two significantly different scales and frequencies, and suspend the particles in different media. All potential and force values were normalized by the primary counterpart to be able to directly compare the results for the different cases.

### 3.1. Polystyrene Particle in Air

**Figure 4:**
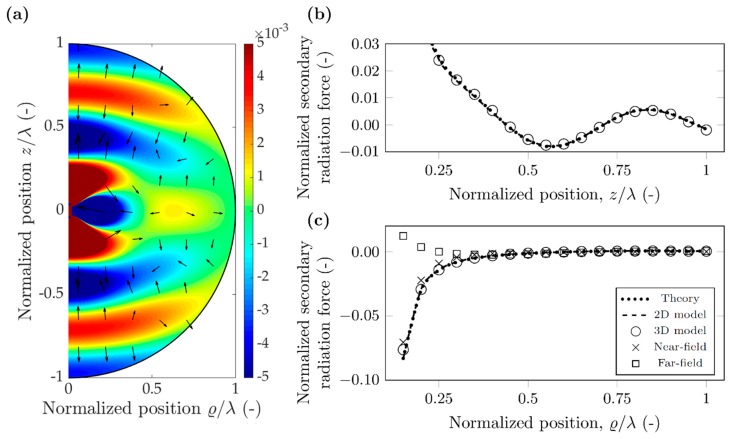
Simulation results for a polystyrene particle in air, when the nodal line aligns with the scatterer position ($h_{n}=0$). (**a**) Normalized secondary acoustic potential, and radiation force (arrows in logarithmic scale) (**b**) Normalized secondary radiation force along direction *z*. (**c**) Normalized secondary radiation force along direction ϱ.

**Figure 5:**
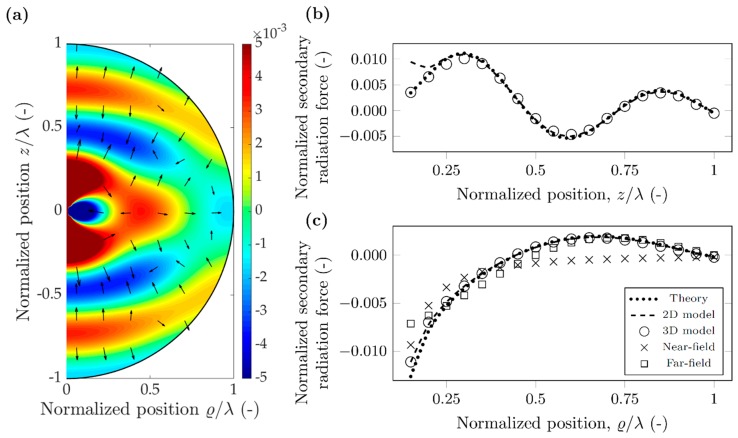
Simulation results for a polystyrene particle in air, when the antinodal line aligns with the scatterer position ($h_{n}=\lambda /4$). (**a**) Normalized secondary acoustic potential, and radiation force (arrows in logarithmic scale) (**b**) Normalized secondary radiation force along direction *z*. (**c**) Normalized secondary radiation force along direction ϱ.

As a first case, we investigated polystyrene (PS) particles in air with the parameters shown in Table 1. The selected 10 kHz frequency is of the same order of that found in acoustic levitation devices [34], and results in wavelength of 34.3 mm in air. Both the scatterer and probe particles are 1.715 mm in diameter, as they were chosen to have diameter *λ*/20 for direct comparison with the case detailed in the Section 3.2 For polystyrene particles in air, the density of the particle is much larger than the density of the surrounding air ($\rho_{\mathrm{PS}}=1050\;\mathrm{kg}/m^{3}\gg 1.225\;\mathrm{kg}/m^{3}=\rho_{\mathrm{AIR}}$), and according to Equation (4b) the dipole scattering factor is approximately unity ($f_{1}\approx 1$). Similarly, the adiabatic compressibility of the air is much larger than the compressibility of the polystyrene particle ($\kappa_{\mathrm{PS}}=172{\;\mathrm{TPa}}^{-1}\ll 694{\;\mathrm{MPa}}^{-1}=\kappa_{\mathrm{AIR}}$), and therefore the particle can be taken as rigid in this case, with monopole scattering coefficient close to unity ($f_{0}\approx 1$), according to Equation (4a). To observe the effect of monopole and dipole coefficients, we show the normalized potential and force values both at the nodal line (*h*_n_ = 0) in Figure 4 and in Figure 5 for the antinodal case (*h*_n_ = *λ*/4). The monopole scattering dominates the secondary force near the antinode, while the dipole scattering dominates near the node [17]. The potential maps in Figure 4a and Figure 5a were plotted using Equation (12), and the arrows show the direction of the force, which points away from the minima, towards the maxima, as the particles have positive contrast factor Φ_AC_. The force was obtained by numerical differentiation, and a logarithmic scaling was used for plotting.

In the two cases, the normalized potential map has a similar pattern and magnitude. This is due to both the monopole and dipole scattering coefficients being approximately unity for a polystyrene particle in air, which can be considered rigid. The normalized secondary radiation force follows similar behavior along the *z* direction and *r* direction for the nodal and antinodal cases, the only significant difference observed for the near-field of the *z* direction. Moreover, it shows some directivity as in the *z* direction being a magnitude stronger than along the radial direction.

Good agreement between theory, 2D model and 3D model can be observed for both nodal and antinodal cases, in both directions, however, the 2D model fails to capture the magnitude of the interaction force in the vicinity of the antinodal line, along *z* direction (Figure 5b). We note here that the three results (theory, 2D and 3D) include different approximations and therefore differences are expected. The theoretical results consider only monopole and dipole scattering mechanisms; however, quadrupole or higher order scattering are important as well [27]. The 2D model does not put a limitation on the number of multipoles but neglects re-scattering effects. Finally, the 3D model, captures arbitrary number of poles (depending on the fineness of discretization) and also accounts for re-scattering. For the radial direction, near the antinodal line, the error between the near-field approximation and the theoretical values is below 50% for normalized distances of less than 0.3. The far-field approximation converges much faster towards the theoretical solution: less than 12% error for normalized distances above 0.65. For the nodal line, the error between the near-field approximation and theoretical values is less than 20% for normalized distances below 0.3. The error of far-field approximation goes below 10% when the normalized distance is larger than 0.65.

The secondary radiation force along the radial direction can be large enough to influence the relative position of the particles. It has a crossover point around 0.63*λ* separation distance along the nodal line; particles closer than this exhibit an attractive (negative) force, while above the crossover point, the force is repulsive (positive). Due to this sign distribution of the force, the 0.63*λ* point is an unstable equilibrium, particles are always forced to move away from it.

As a summary, for the rigid particle in air, the two simulation results are in good agreement with the theory, except for forces along the *z* direction near an antinodal line. However, as the particles naturally agglomerate at the nodes, the secondary radiation force can be obtained using a simplified model and neglecting re-scattering effects.

### 3.2. Polystyrene Particle in Water

**Figure 6:**
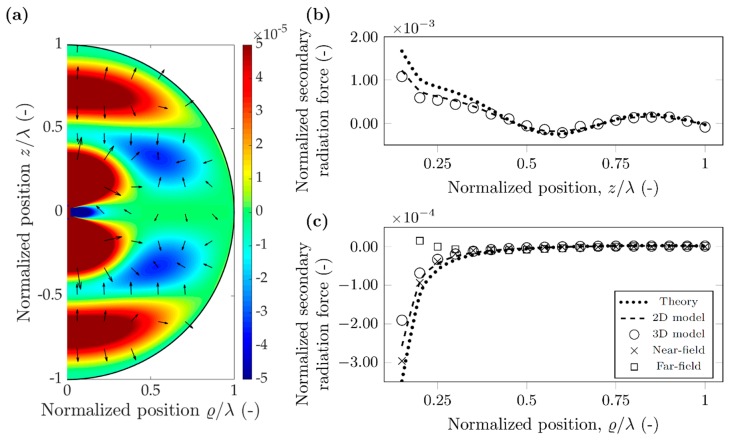
Simulation results for a polystyrene particle in water, when the nodal line aligns with the scatterer position ($h_{n}=0$). (**a**) Normalized secondary acoustic potential, and radiation force (arrows in logarithmic scale) (**b**) Normalized secondary radiation force along direction *z*. (**c**) Normalized secondary radiation force along direction ϱ.

**Figure 7:**
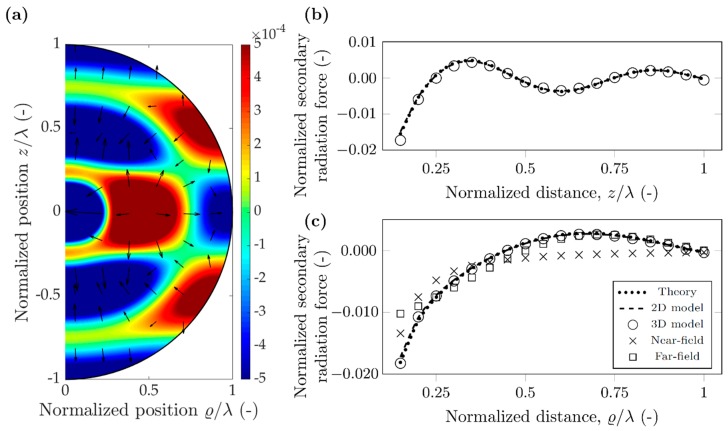
Simulation results for a polystyrene particle in water, when the antinodal line aligns with the scatterer position ($h_{n}=\lambda /4$). (**a**) Normalized secondary acoustic potential, and radiation force (arrows in logarithmic scale) (**b**) Normalized secondary radiation force along direction *z*. (**c**) Normalized secondary radiation force along direction ϱ.

A different investigation can be carried out when placing the polystyrene particle in water. As the main goal of microfluidic lab-on-chip devices is miniaturization, the operating frequency has to be increased, therefore the chosen 10 MHz reflects this typical average value [3,37]. For further parameters, refer to Table 2. The resulting wavelength is 148 µm, and the particle diameter 7.4 µm. More interesting to note the change in the scattering coefficients: the similarity in compressibilities of the particle and water ($\kappa_{\mathrm{PS}}=172\;{\mathrm{TPa}}^{-1}$, $\kappa_{\mathrm{WATER}}=456\;{\mathrm{TPa}}^{-1}$) results in $f_{0}=0.623$. More significant is the drop in the dipole scattering coefficient: due to the similarity of the densities (0.998 and 1.05 g/cm^3^), $f_{1}\approx 0.03\ll 1$. The potential maps in Figure 6a and Figure 7a were again plotted using Equation (12), and the arrows show the direction of the force, again pointing away from the minima towards the maxima, as the particles have positive contrast factor *Φ_AC_*.

For the polystyrene particle in water, the potential map around the node and the antinode has a significantly different shape and magnitude. This is due to the difference in monopole and dipole scattering coefficient [17]. Referring to Equation (12), each term of the potential has either a dipole coefficient, *f*_1_, or a sin(*kh*_n_) expression. At the nodes sin(*kh*_n_) is zero, and the dipole coefficient is much less than unity, as mentioned before, leading to the potential an order of magnitude smaller near the nodes compared to the antinodes. This difference is even more pronounced for the force along the radial direction: here the difference exceeds two orders of magnitude. Along *z* direction, the force is repulsive for the nodes, attractive for the antinodes, but again as its magnitude is much smaller than the primary force, no effect on particles is expected.

For the antinodal case excellent agreement between theory and the two types of simulations can be observed. This shows that the theoretically assumed monopole and dipole approximation already successfully captures the secondary radiation force with small error. Furthermore, the good agreement between 2D and 3D models suggest that in this case, the re-scattering effects also contribute only slightly to the secondary radiation force. For the radial direction, the near-field approximation shows similar performance as for polystyrene in air, the error goes below 50% only when the normalized distances are less than 0.3. The far-field approximation shows slower convergence in this case: the error is 40% even at 0.85 distance.

However, for the nodal case, the three results can be different up to 50% error. The magnitude of the theoretical values is the largest, followed by the 2D model and the 3D model, seemingly the re-scattering events decrease the secondary force, as also noted by Doinikov for a bubble in water case [27]. The near-field and far-field approximations shown good agreement with the various models and the theory (Figure 6c).

Although the particles both in air and water agglomerate at the nodal lines (Φ_AC_ > 0), we believe it is important to investigate the secondary radiation force for other cases. In a continuous flow microfluidic device the particles enter at random positions and can be near an antinodal line when they first experience the acoustic field. In addition, as Figure 7 suggests, in this case the attractive secondary force (which is two orders of magnitude higher than around the nodal line) can trap particles together, negatively affecting device performance [38].

### 3.3. Properties of the Secondary Radiation Force

**Figure 8:**
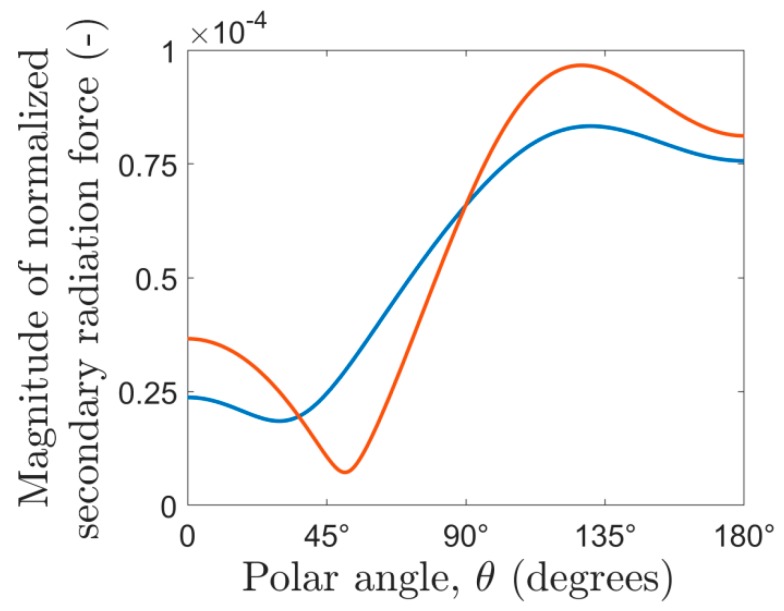
The magnitude of the secondary radiation force on the probe particle (blue) and on the scatterer particle (red) as a function of the polar angle (*θ*). For an antisymmetric force, the magnitude on the scatterer and the probe would be the same for any polar angle.

It is important to emphasize two properties of the secondary radiation force: it is neither antisymmetric nor central in a general case. As the two acoustic fields re-scattered by the source and probe particles are usually different (unless they are positioned such that the background pressure field is in phase at these locations), the effect of the two particles on each other is not interchangeable, or the secondary radiation force is not antisymmetric. To illustrate this, we used two polystyrene particles in water (with the parameters presented in Table 2 and *h* = 20 µm) and plotted the magnitude of the secondary radiation force on both particles, while we moved the probe particle on an arc of radius λ/5, from θ=0 to θ=π. To obtain the secondary radiation force on the scatterer particle (i.e., when the roles of the two particles are interchanged) we shifted both the particles and the external field by −λ/5cosθ in the *z*, and by −λ/5sinθ in the ϱ direction, so in this case the probe particle was located at the origin and acted as if it was the scatterer. As Figure 8 shows, the magnitude of the two forces differ at each point of the arc, except the two intersection points of the curves.

**Figure 9:**
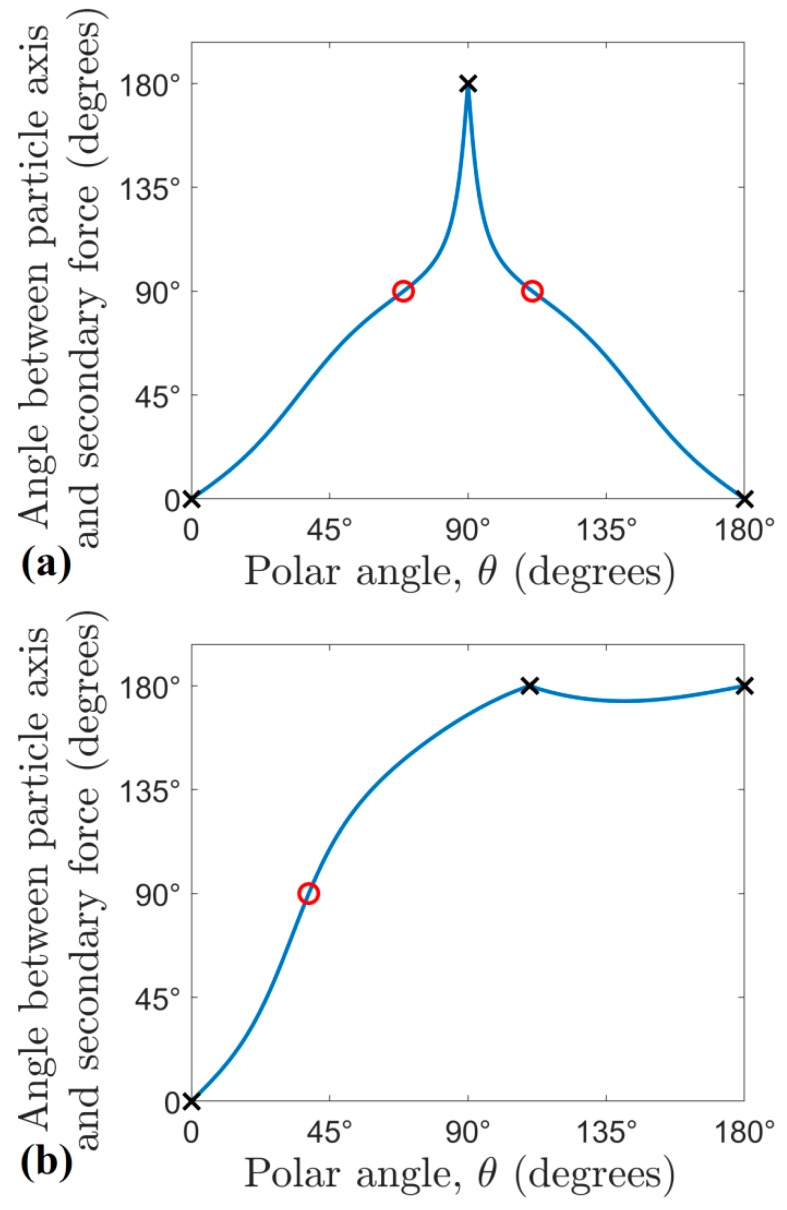
The angle between the secondary radiation force and the axis of the particles, as a function of the polar angle (*θ*), (**a**) corresponds to *h*_n_ = 0 µm, (**b**) shows *h*_n_ = 20 µm case. This angle should either be zero or *π*, when the force is perfectly central. These points are marked with crosses. Circles represent perpendicular secondary forces to the particle axis.

To show that the secondary radiation force is not central, i.e., the force does not usually act along the axis of the two particles, we performed the following examination: the probe particle was again moved on the same arc as before, and now the angle between the secondary force and the radial unit vector was recorded. For central forces, this angle is either zero or *π*, depending on whether the force is repulsive or attractive. However, as shown in Figure 9, the force acts along the axis of the particles only at three locations (marked by crosses). In general, the secondary force is of arbitrary angle with respect to the axis of the particles, and can be even perpendicular to it (marked by circles).

Specifically, for *h*_n_ = 20 µm (Figure 9b), as the scatterer is not at either the nodes or antinodes (h≠0 and h≠λ/4), the force does not even act along the axis of the particles in the transversal plane, but has a polar non-zero component (for θ=π/2 the angle is neither zero or *π*).

The same test was also carried out when the scatterer particle was aligned with the natural trapping pressure node (*h*_n_ = 0, Figure 9a). In this case, the force is central when the probe particle is along the transversal plane (*θ* = *π*/2), as shown by the cross in the middle of Figure 9a. We marked again the positions corresponding to perpendicular secondary force to the particle axis by red circles.

## 4. Discussion and Conclusions

In this paper, we presented two finite element models to obtain the secondary radiation force between spheres suspended in air or water. For the polystyrene particle in air, the investigation revealed no significant difference between the analytical and numerical approaches. However, for polystyrene particle in water, the difference between theory and models can be up to 50%. As the analytical equation results in the largest magnitude of force, it can be used for safe overestimation of the secondary interaction force. The 3D model, including re-scattering between the two particles, showed no difference to the simpler 2D model for most cases; therefore, the 2D axisymmetric model can be used for fast determination of acoustic radiation force, with sufficient precision. The only significant difference was for the polystyrene particle in water case, when the scatterer is aligned with the node, forces along both the *r-* and *z*-axis. Here all the theoretical, 2D and 3D models showed different results with up to 50% variance, indicating the significance of the re-scattering effects in these cases. Moreover, for the polystyrene in air, scatterer at the antinode case, a similar, but less pronounced difference could be seen. For this case, along the *z* axis, the 2D model was not able to capture accurately the radiation force for small separation distances (<λ/4); a full 3D model for small distances is required. When there are only a few particles to be investigated, with large separation distances, even the 2D simulation can be swapped for the simpler analytical pairwise force determination.

The advantage of both these techniques is the easy implementation and simple adjustment to parameter change. Moreover, the numerical method based on the tensor integral is not restricted to particles much smaller than the acoustic wavelength and can be applied to particles with arbitrary shapes and sizes. Computational requirement to achieve 1% convergence error or less was in the order of seconds for the 2D model and in the order of tens of seconds for the 3D model. The presented models can be complementary when theoretical equations are difficult to obtain, for example arbitrary shaped bodies. Moreover, although the models were verified for a plane standing wave field they can be easily extended to calculate the interacting forces in an arbitrary acoustic field.

A limitation of the technique that it cannot be applied directly for touching spheres, but the surface-to-surface separation can be as low as 0.001*λ* for successful simulation. Viscous effects can be incorporated within the model using a thermoviscous acoustics module for the acoustic field simulations [40]. Moreover, investigation of streaming effects is possible by extending the model with a laminar flow module for simulation of streaming velocity fields [36,41].

## Figures and Tables

**Table 1:** Parameters used for the simulation of rigid particle in air case.

Symbol	Description	Value
*f*	Frequency	10 kHz
*c*_0_	Speed of sound in air ^a^	343 m/s
*λ*	Wavelength in air	34.3 mm
*ρ*_0_	Density of air	1.225 kg/m^3^
*a*_s_, *a*_p_	Radius of scatterer and probe particle (λ/20)	1.715 mm
*ρ*_s_, *ρ*_p_	Density of scatterer and probe particle ^b^	1050 kg/m^3^
*c*_s_, *c*_p_	Speed of sound of scatterer and probe ^b^	2350 m/s
*f*_0,s_, *f*_0,p_	Monopole scattering coefficient ^c^	0.99998
*f*_1,s_, *f*_1,p_	Dipole scattering coefficient ^d^	0.99825
*p*_0_	Acoustic pressure amplitude	50 kPa
Φ_AC_	Acoustic contrast factor	2.4974

^a^ From [35]. ^b^ From [36]. ^c,d^ Calculated according to Equations (4a) and (4b).

**Table 2:** Parameters used for the simulation of a polystyrene particle in water case.

Symbol	Description	Value
*f*	Frequency	10 MHz
*c*_0_	Speed of sound in water ^a^	1480 m/s
*λ*	Wavelength in air	148 µm
*ρ*_0_	Density of water	998 kg/m^3^
*a*_s_, *a*_p_	Radius of scatterer and probe particle (λ/20)	7.4 µm
*ρ*_s_, *ρ*_p_	Density of scatterer and probe particle ^b^	1050 kg/m^3^
*c*_s_, *c*_p_	Speed of sound of scatterer and probe ^b^	2350 m/s
*f*_0,s_, *f*_0,p_	Monopole scattering coefficient ^c^	0.623
*f*_1,s_, *f*_1,p_	Dipole scattering coefficient ^d^	0.034
*p*_0_	Acoustic pressure amplitude	50 kPa
Φ_AC_	Acoustic contrast factor	0.6734

^a^ From [39]. ^b^ From [36]. ^c,d^ Calculated according to Equations (4a) and (4b).

## Supplementary Materials

The following are available online at https://www.mdpi.com/2072-666X/10/7/431/s1, Figure S1: Convergence analysis results when the scattering particle is aligned with the pressure antinode and the probe particle is along the *z* direction; Figure S2: Convergence analysis results when the scattering particle is aligned with the pressure node and the probe particle is along the *z* direction; Figure S3: Convergence analysis results when the scattering particle is aligned with the pressure antinode and the probe particle is along the *r* direction; Figure S4: Convergence analysis results when the scattering particle is aligned with the pressure node and the probe particle is along the *r* direction; Figure S5: Convergence analysis results when the scattering particle is aligned with the pressure node and the probe particle is along either the *r* or *z* direction; Figure S6: Convergence analysis results when the scattering particle is aligned with the pressure antinode and the probe particle is along either the *r* or *z* direction; Figure S7: Secondary radiation force when the scattering particle is aligned with the pressure antinode and the probe particle is along the *z* direction; Table S1: Relation between number of degrees of freedom and mesh scale parameter for the 2D mesh convergence analysis.
