# Supplementary material for: Numerical Determination of the Secondary Acoustic Radiation Force on a Small Sphere in a Plane Standing Wave Field
Source: Micromachines (Basel). 2019 Jun 29;10(7):431. doi: 10.3390/mi10070431 (PMC6680761; doi:10.3390/mi10070431)
Supplement: Supplementary file 1 [file micromachines-10-00431-s001.pdf]

Supplementary Materials: Numerical Determination of the Secondary Acoustic Radiation Force on a Small Sphere in a Plane Standing Wave Field

Gergely Simon [1,2], Marco A. B. Andrade [3], Marc P. Y. Desmulliez [1], Mathis O. Riehle [4] and Anne L. Bernassau [1,*]

[1] School of Engineering and Physical Sciences, Heriot-Watt University, Edinburgh EH14 4AS, U.K.
[2] OnScale Ltd., Glasgow, G2 5QR, U.K.
[3] Institute of Physics, University of São Paulo, 05508-090, São Paulo, Brazil
[4] Institute of Molecular Cell and Systems Biology, Centre for Cell Engineering, University of Glasgow, Glasgow G12 8QQ, U.K.
* Correspondence: a.bernassau@hw.ac.uk

Primary radiation force potential

To obtain Equation (8) in the main body of the article, the following steps can be performed:

$$U_{\mathrm{prim}}(\boldsymbol{r}) = \frac{V_i}{2}\left[\frac{1}{2} f_{0,i}\kappa_0 (c_0\rho_0 v_0)^2 \sin^2[k(z-h_{\mathrm{n}})] - \frac{3}{4} f_{1,i}\rho_0 v_0^2 \cos^2[k(z-h_{\mathrm{n}})]\right]$$

$$U_{\mathrm{prim}}(\boldsymbol{r}) = \frac{V_i\rho_0 v_0^2}{2}\left[\frac{1}{2} f_{0,i} \sin^2[k(z-h_{\mathrm{n}})] - \frac{3}{4} f_{1,i} \cos^2[k(z-h_{\mathrm{n}})]\right]$$

and using the identity $\sin^2 x + \cos^2 x = 1$ we have

$$U_{\mathrm{prim}}(\boldsymbol{r}) = \frac{V_i E_0}{2}\left[f_{0,i} - \left(f_{0,i} + \frac{3}{2} f_{1,i}\right)\cos^2[k(z-h_{\mathrm{n}})]\right] = \frac{V_i E_0}{2}\left[f_{0,i} - \Phi_{\mathrm{AC}} \cos^2[k(z-h_{\mathrm{n}})]\right]$$

Secondary radiation force potential

The derivation of this section follows the steps of Silva and Bruus.

According to Gorkov's potential theory, the acoustic radiation potential of any arbitrary field, except a plane travelling wave, can be obtained as:

$$U(\boldsymbol{r}_{\mathrm{p}}) = a_{\mathrm{p}}^3 \pi \rho_0 \left[\frac{f_{0,\mathrm{p}}}{3} k^2 \left|\phi_{\mathrm{total}}(\boldsymbol{r}_{\mathrm{p}})\right|^2 - \frac{f_{1,\mathrm{p}}}{2}\left|\nabla_{\mathrm{p}}\phi_{\mathrm{total}}(\boldsymbol{r}_{\mathrm{p}})\right|^2\right] \tag{S1}$$

Moreover, in our case, the total velocity potential is the sum of the velocity potential of the external field and the rescattered field:

$$\phi_{\mathrm{total}}(\boldsymbol{r},t) = \phi_{\mathrm{ext}}(\boldsymbol{r},t) + \phi_{\mathrm{sc}}(\boldsymbol{r},t) \tag{S2}$$

For complex fields

$$\left|\phi_{\mathrm{total}}(\boldsymbol{r}_{\mathrm{p}})\right|^2 = \left|\phi_{\mathrm{ext}}(\boldsymbol{r}_{\mathrm{p}}) + \phi_{\mathrm{sc}}(\boldsymbol{r}_{\mathrm{p}})\right|^2 = \left(\phi_{\mathrm{ext}}(\boldsymbol{r}_{\mathrm{p}}) + \phi_{\mathrm{sc}}(\boldsymbol{r}_{\mathrm{p}})\right)^* \left(\phi_{\mathrm{ext}}(\boldsymbol{r}_{\mathrm{p}}) + \phi_{\mathrm{sc}}(\boldsymbol{r}_{\mathrm{p}})\right) = \phi_{\mathrm{ext}}(\boldsymbol{r}_{\mathrm{p}})^*\phi_{\mathrm{ext}}(\boldsymbol{r}_{\mathrm{p}}) + \phi_{\mathrm{ext}}(\boldsymbol{r}_{\mathrm{p}})^*\phi_{\mathrm{sc}}(\boldsymbol{r}_{\mathrm{p}}) + \phi_{\mathrm{sc}}(\boldsymbol{r}_{\mathrm{p}})^*\phi_{\mathrm{ext}}(\boldsymbol{r}_{\mathrm{p}}) + \phi_{sc}(\boldsymbol{r}_{\mathrm{p}})^*\phi_{sc}(\boldsymbol{r}_{\mathrm{p}}) \tag{S3}$$

Here the first term corresponds to the primary radiation potential, the last term is small compared to the second and third terms. Moreover,

$$\mathrm{Re}[a^* b] = \mathrm{Re}[a b^*] \tag{S4a}$$

and therefore the second and third terms can be contracted

$$\phi_{\mathrm{ext}}(\boldsymbol{r}_{\mathrm{p}})^* \phi_{\mathrm{sc}}(\boldsymbol{r}_{\mathrm{p}}) + \phi_{\mathrm{sc}}(\boldsymbol{r}_{\mathrm{p}})^* \phi_{\mathrm{ext}}(\boldsymbol{r}_{\mathrm{p}}) = 2\mathrm{Re}\left[\phi_{\mathrm{ext}}(\boldsymbol{r}_{\mathrm{p}})^* \phi_{\mathrm{sc}}(\boldsymbol{r}_{\mathrm{p}})\right] \tag{S4b}$$

and the potential of the secondary acoustic field can be approximated as

$$U_{\mathrm{sec}}(\boldsymbol{r}_{\mathrm{p}}) = a_{\mathrm{p}}^3 \pi \rho_0 \left[\frac{f_{0,\mathrm{p}}}{3} k^2 \mathrm{Re}\left[\phi_{\mathrm{ext}}^*(\boldsymbol{r}_{\mathrm{p}}) \phi_{\mathrm{sc}}(\boldsymbol{r}_{\mathrm{p}}|\boldsymbol{r}_{\mathrm{s}})\right] - \frac{f_{1,\mathrm{p}}}{2} \mathrm{Re}\left[\nabla_{\mathrm{p}}^2 \phi_{\mathrm{ext}}^*(\boldsymbol{r}_{\mathrm{p}}) \phi_{\mathrm{sc}}(\boldsymbol{r}_{\mathrm{p}}|\boldsymbol{r}_{\mathrm{s}})\right]\right] \tag{S5a}$$

or equivalently

$$U_{\mathrm{sec}}(\boldsymbol{r}_{\mathrm{p}}|\boldsymbol{r}_{\mathrm{s}}) = \pi k^2 a_{\mathrm{p}}^3 \rho_0 \mathrm{Re}\left[\frac{2 f_{0,\mathrm{p}}}{3} \phi_{\mathrm{ext}}^*(\boldsymbol{r}_{\mathrm{p}}) \phi_{\mathrm{sc}}(\boldsymbol{r}_{\mathrm{p}}|\boldsymbol{r}_{\mathrm{s}}) - f_{1,\mathrm{p}} \frac{1}{k} \nabla_{\mathrm{p}} \phi_{\mathrm{ext}}^*(\boldsymbol{r}_{\mathrm{p}}) \cdot \frac{1}{k} \nabla_{\mathrm{p}} \phi_{\mathrm{sc}}(\boldsymbol{r}_{\mathrm{p}}|\boldsymbol{r}_{\mathrm{s}})\right] = U_{\mathrm{sec},0} - U_{\mathrm{sec},1} \tag{S5b}$$

The scattered wave due to an external field can be given as

$$\phi_{\mathrm{sc}}(\boldsymbol{r}_{\mathrm{p}}|\boldsymbol{r}_{\mathrm{s}}) = i f_{0,\mathrm{s}} \frac{a_{\mathrm{s}}^3 \omega}{3 \rho_0} \frac{\rho_{\mathrm{in}}(\boldsymbol{r}_{\mathrm{s}}) e^{i k r_{\mathrm{sp}}}}{r_{\mathrm{sp}}} - f_{1,\mathrm{s}} \frac{a_{\mathrm{s}}^3}{2} \nabla_{\mathrm{p}} \cdot \left[\frac{\boldsymbol{v}_{\mathrm{in}}(\boldsymbol{r}_{\mathrm{s}}) e^{i k r_{\mathrm{sp}}}}{r_{\mathrm{sp}}}\right] + O\left[\frac{(k a_{\mathrm{s}})^5}{(k r_{\mathrm{sp}})^3}\right] \tag{S6}$$

where

$$\rho_{\mathrm{in}}(\boldsymbol{r}) = i \frac{\omega \rho_0}{c_o^2} \phi_{\mathrm{in}}(\boldsymbol{r}) \tag{S7a}$$

and

$$\boldsymbol{v}_{\mathrm{in}}(\boldsymbol{r}) = \nabla \phi_{\mathrm{in}}(\boldsymbol{r}) \tag{S7b}$$

Direct substitution of the velocity field and density gives the scattered velocity potential. To ease the treatment of the scattered velocity potential, we denote the two separate terms with index 0 and 1, and neglect the argument $(\boldsymbol{r}_p|\boldsymbol{0})$ in the following

$$\phi_{\mathrm{sc}}(\boldsymbol{r}_{\mathrm{p}}|\boldsymbol{0}) = \phi_{\mathrm{sc},0}(\boldsymbol{r}_{\mathrm{p}}|\boldsymbol{0}) - \phi_{\mathrm{sc},1}(\boldsymbol{r}_{\mathrm{p}}|\boldsymbol{0}) \tag{S8}$$

Since the velocity potential of the external field is a real number, its complex conjugate is itself

$$\phi_{\mathrm{ext}} = \phi_{\mathrm{ext}}^* = \frac{v_0}{k} \sin[k(r \cos\theta - h_{\mathrm{n}})] \tag{S9}$$

and therefore, its gradient is

$$\nabla_{\mathrm{p}} \phi_{\mathrm{ext}}^*(\boldsymbol{r}_{\mathrm{p}}) = v_0 \cos[k(r \cos\theta - h_{\mathrm{n}})] \{\cos\theta\, \hat{\boldsymbol{r}} - \sin\theta\, \hat{\boldsymbol{\theta}}\} \tag{S10}$$

Therefore, the first term of Equation (S8) is

$$\phi_{\mathrm{sc},0} = i f_{0,\mathrm{s}} \frac{a_{\mathrm{s}}^3 \omega}{3 \rho_0} \frac{\rho_{\mathrm{in}}(\boldsymbol{0}) e^{i k r_{\mathrm{sp}}}}{r_{\mathrm{sp}}} = f_{0,\mathrm{s}} \frac{a_{\mathrm{s}}^3 k^2}{3} \frac{v_0 \sin k h_{\mathrm{n}} e^{i k r_{\mathrm{sp}}}}{k r_{\mathrm{sp}}} \tag{S11a}$$

and its real part

$$\mathrm{Re}[\phi_{\mathrm{sc},0}] = f_{0,\mathrm{s}} \frac{a_{\mathrm{s}}^3 k^2}{3} \frac{v_0 \sin k h_{\mathrm{n}} \cos k r_{\mathrm{sp}}}{k r_{\mathrm{sp}}} \tag{S11b}$$

For the second term of Equation (S8), first

$$\nabla_\mathrm{p} \cdot \left[\frac{v_\mathrm{in}(\boldsymbol{r}_\mathrm{s}) e^{ikr_\mathrm{sp}}}{r_\mathrm{sp}}\right] = v_0 \cos\theta \cos kh_\mathrm{n} \left[\frac{1}{r_\mathrm{sp}^2} + \frac{ik}{r_\mathrm{sp}}\right] e^{ikr_\mathrm{sp}} - \frac{2v_0 \cos\theta \cos kh_\mathrm{n}}{r_\mathrm{sp}^2} e^{ikr_\mathrm{sp}} =$$
$$v_0 \cos\theta \cos kh_n \left[\frac{ik}{r_{sp}} - \frac{1}{r_{sp}^2}\right] e^{ikr_{sp}} \quad \text{(S12a)}$$

such that the second term of Equation (S8) is

$$\phi_\mathrm{sc,1}(\boldsymbol{r}_\mathrm{p}|\boldsymbol{0}) = f_\mathrm{1,s} \frac{a_\mathrm{s}^3}{2} v_0 \cos\theta \cos kh_\mathrm{n} \left[\frac{ik}{r_\mathrm{sp}} - \frac{1}{r_\mathrm{sp}^2}\right] e^{ikr_\mathrm{sp}} \quad \text{(S12b)}$$

Its real part being

$$\mathrm{Re}[\phi_\mathrm{sc,1}] = -f_\mathrm{1,s} \frac{a_\mathrm{s}^3}{2} k^2 v_0 \cos\theta \cos kh_\mathrm{n} \left(\frac{\sin kr_\mathrm{sp}}{kr_\mathrm{sp}} + \frac{\cos kr_\mathrm{sp}}{(kr_\mathrm{sp})^2}\right) \quad \text{(S12c)}$$

As the first term of the secondary radiation potential, Equation (S5b), depends on the velocity potential of the external field, which is real, and the real parts of the scattered velocity potential are given by Equations (S11b) and (S12c), $U_\mathrm{sec,0}$ can be obtained:

$$U_\mathrm{sec,0} = \pi k^2 a_\mathrm{p}^3 \rho_0 \mathrm{Re}\left[\frac{2f_\mathrm{0,p}}{3} \phi_\mathrm{ext}^* \phi_\mathrm{sc}\right] = \pi k^2 a_\mathrm{p}^3 \rho_0 \frac{2f_\mathrm{0,p}}{3} \phi_\mathrm{ext}^* \mathrm{Re}[\phi_\mathrm{sc,0} - \phi_\mathrm{sc,1}] =$$
$$\pi k^2 a_\mathrm{p}^3 \rho_0 \frac{2f_\mathrm{0,p}}{3} \frac{v_0}{k} \sin[k(r\cos\theta - h_\mathrm{n})] \left\{ f_\mathrm{0,s} \frac{a_\mathrm{s}^3 k^2}{3} \frac{v_0 \sin kh_\mathrm{n} \cos kr_\mathrm{sp}}{kr_\mathrm{sp}} + \right.$$
$$\left. f_\mathrm{1,s} \frac{a_\mathrm{s}^3}{2} k^2 v_0 \cos\theta \cos kh_\mathrm{n} \left(\frac{\sin kr_\mathrm{sp}}{kr_\mathrm{sp}} + \frac{\cos kr_\mathrm{sp}}{(kr_\mathrm{sp})^2}\right)\right\} = \frac{1}{2} \pi k^3 a_\mathrm{p}^3 a_\mathrm{s}^3 \rho_0 v_0^2 \frac{2f_\mathrm{0,p}}{3} \sin[k(r\cos\theta -$$
$$h_\mathrm{n})] \left\{\frac{2}{3} f_{0,s} \frac{\sin kh_\mathrm{n} \cos kr_\mathrm{sp}}{kr_\mathrm{sp}} + f_\mathrm{1,s} \cos\theta \cos kh_\mathrm{n} \left(\frac{\sin kr_\mathrm{sp}}{kr_\mathrm{sp}} + \frac{\cos kr_\mathrm{sp}}{(kr_\mathrm{sp})^2}\right)\right\} \quad \text{(S13)}$$

The second term of the secondary potential, Equation (S5b), can be calculated by splitting the scattered potential:

$$U_\mathrm{sec,1} = \pi a_\mathrm{p}^3 \rho_0 f_\mathrm{1,p} \mathrm{Re}[\nabla_\mathrm{p} \phi_\mathrm{ext}^*(\boldsymbol{r}_\mathrm{p}) \cdot \nabla_\mathrm{p} \phi_\mathrm{sc}] \quad \text{(S14a)}$$

$$\mathrm{Re}[\nabla_\mathrm{p} \phi_\mathrm{ext}^*(\boldsymbol{r}_\mathrm{p}) \cdot \nabla_\mathrm{p} \phi_\mathrm{sc}] = \mathrm{Re}[\nabla_\mathrm{p} \phi_\mathrm{ext}^*(\boldsymbol{r}_\mathrm{p}) \cdot \nabla_\mathrm{p} \phi_\mathrm{sc,0}] - \mathrm{Re}[\nabla_\mathrm{p} \phi_\mathrm{ext}^*(\boldsymbol{r}_\mathrm{p}) \cdot \nabla_\mathrm{p} \phi_\mathrm{sc,1}] \quad \text{(S14b)}$$

Equation (S14b), requires the calculation of some auxiliary terms (the gradient of the scattered velocity potential):

$$\nabla_\mathrm{p} \phi_\mathrm{sc,0}(\boldsymbol{r}_\mathrm{p}|\boldsymbol{0}) = f_\mathrm{0,s} \frac{a_\mathrm{s}^3 k}{3} v_0 \sin kh_\mathrm{n} \left[\frac{ikr_\mathrm{sp} - 1}{r_\mathrm{sp}^2}\right] e^{ikr_\mathrm{sp}} \hat{\boldsymbol{r}} \quad \text{(S15a)}$$

$$\nabla_\mathrm{p} \phi_\mathrm{sc,1}(\boldsymbol{r}_\mathrm{p}|\boldsymbol{0}) = f_\mathrm{1,s} \frac{a_\mathrm{s}^3}{2} v_0 \cos kh_\mathrm{n} \frac{e^{ikr_\mathrm{sp}}}{r_\mathrm{sp}^3} \left\{-\cos\theta \left((kr_\mathrm{sp})^2 + 2ikr_\mathrm{sp} - 2\right) \hat{\boldsymbol{r}} - \sin\theta \left(ikr_\mathrm{sp} - 1\right) \hat{\boldsymbol{\theta}}\right\}$$
(S15b)

and consequently, the first term of Equation (S14b)

$$\nabla_\mathrm{p} \phi_\mathrm{ext}^*(\boldsymbol{r}_\mathrm{p}) \cdot \nabla_\mathrm{p} \phi_\mathrm{sc,0} = v_0 \cos\theta \cos[k(r\cos\theta - h_\mathrm{n})] f_\mathrm{0,s} \frac{a_\mathrm{s}^3 k}{3} v_0 \sin kh_\mathrm{n} \left[\frac{ik\ \ \mathrm{sp} - 1}{r_\mathrm{sp}^2}\right] e^{ik\ \ \mathrm{sp}} \quad \text{(S16a)}$$

with its real part

$$\mathrm{Re}\big[\nabla_\mathrm{p}\phi^*_\mathrm{ext}(\boldsymbol{r}_\mathrm{p})\cdot\nabla_\mathrm{p}\phi_\mathrm{sc,0}\big] = -v_0^2 f_\mathrm{0,s}\frac{a_\mathrm{s}^3k^3}{3}\cos[k(r\cos\theta-h_\mathrm{n})]\cos\theta\sin kh_\mathrm{n}\left\{\frac{\sin kr}{kr}+\frac{\cos kr}{(kr)^2}\right\} \quad \text{(S16b)}$$

and the second term of Equation (S14b)

$$\nabla_\mathrm{p}\phi^*_\mathrm{ext}(\boldsymbol{r}_\mathrm{p})\cdot\nabla_\mathrm{p}\phi_\mathrm{sc,1} = v_0\cos[k(r\cos\theta-h_\mathrm{n})]\,f_\mathrm{1,s}\frac{a_\mathrm{s}^3}{2}v_0\cos kh_\mathrm{n}\frac{e^{ikr_\mathrm{sp}}}{r_\mathrm{sp}^3}\left\{-\cos^2\theta\left(\left(kr_\mathrm{sp}\right)^2+2ikr_\mathrm{sp}-2\right)+\sin^2\theta\left(ikr_\mathrm{sp}-1\right)\right\} \quad \text{(S17a)}$$

with its real part

$$\mathrm{Re}\big[\nabla_\mathrm{p}\phi^*_\mathrm{ext}(\boldsymbol{r}_\mathrm{p})\cdot\nabla_\mathrm{p}\phi_\mathrm{sc,1}\big] = v_0\cos[k(r\cos\theta-h_\mathrm{n})]\,f_\mathrm{1,s}\frac{a_\mathrm{s}^3}{2}v_0\cos kh_\mathrm{n}\left\{-\frac{\cos^2\theta\cos kr}{r_\mathrm{sp}^3}\left(\left(kr_\mathrm{sp}\right)^2-2\right)-\frac{\sin^2\theta\cos kr}{r_\mathrm{sp}^3}+\frac{2kr\sin kr\,\mathrm{co}\ ^2\theta}{r^3}-\frac{kr\sin^2\theta\,\mathrm{si}}{r^3}\right\} \quad \text{(S17b)}$$

after simplification and using

$$2\cos^2\theta-\sin^2\theta=\frac{1+3\cos 2\theta}{2} \quad \text{(S17c)}$$

Equation (S17b) can be written as

$$\mathrm{Re}\big[\nabla_\mathrm{p}\phi^*_\mathrm{ext}(\boldsymbol{r}_\mathrm{p})\cdot\nabla_\mathrm{p}\phi_\mathrm{sc,1}\big] = v_0^2 f_\mathrm{1,s}\frac{a_\mathrm{s}^3}{4}k^3\cos[k(r\cos\theta-h_\mathrm{n})]\cos kh_\mathrm{n}\left\{\frac{\cos kr}{(kr)^3}(1+3\cos 2\theta)-\frac{2\cos^2\theta\cos kr}{kr}+\frac{(1+3\cos 2\theta)\sin kr}{(kr)^2}\right\} \quad \text{(S17d)}$$

At this point everything is known to obtain $U_\mathrm{sec,1}$, by Equations (S16b) and (S17d):

$$U_\mathrm{sec,1} = -v_0^2\pi a_\mathrm{p}^3 a_\mathrm{s}^3\rho_0 f_\mathrm{1,p}k^3\cos[k(r\cos\theta-h_\mathrm{n})]\left[\frac{f_\mathrm{0,s}}{3}\cos\theta\sin kh_\mathrm{n}\left\{\frac{\sin kr}{kr}+\frac{\cos kr}{(kr)^2}\right\}+\frac{f_\mathrm{1,s}}{4}\cos kh_\mathrm{n}\left\{\frac{\cos kr}{(kr)^3}(1+3\cos 2\theta)-\frac{2\cos^2\theta\cos}{kr}+\frac{(1+3\cos 2\theta)\sin kr}{(kr)^2}\right\}\right] \quad \text{(S18)}$$

and now Equations (S5b), (S13) and (S18) yield the secondary radiation potential:

$$U_\mathrm{sec}(r,\theta) = \pi E_0 k^3 a_\mathrm{p}^3 a_\mathrm{s}^3\Big(\cos[k(r\cos\theta-h_\mathrm{n})]\frac{f_\mathrm{1,p}}{2}\Big\{f_\mathrm{1,s}\cos(kh_\mathrm{n})(1+3\cos 2\theta)\frac{\cos kr}{(kr)^3}+\left[\frac{4}{3}f_\mathrm{0,s}\sin(kh_\mathrm{n})\cos\theta\cos kr+f_\mathrm{1,s}\cos(kh_\mathrm{n})(1+3\cos 2\theta)\sin kr\right]\frac{1}{(kr)^2}-\left[f_\mathrm{1,s}\cos(kh_\mathrm{n})(1+\cos 2\theta)\cos kr-\frac{4}{3}f_\mathrm{0,s}\sin(kh_\mathrm{n})\cos\theta\sin kr\right]\frac{1}{kr}\Big\}+\sin[k(r\cos\theta-h_\mathrm{n})]\frac{2f_\mathrm{0,p}}{3}\Big\{f_\mathrm{1,s}\cos(kh_\mathrm{n})\cos\theta\frac{\cos kr}{(kr)^2}+\left[\frac{2}{3}f_\mathrm{0,s}\sin(kh_\mathrm{n})\cos kr+f_\mathrm{1,s}\cos(kh_\mathrm{n})\cos\theta\sin kr\right]\frac{1}{kr}\Big\}\Big) \quad \text{(S19)}$$

Secondary radiation force in the radial direction for a general case

The derivatives of terms containing r:

where [ ] denotes $[k(r\cos\theta-h_n)]$ used for shorter notation

$$\frac{\partial}{\partial r}\cos[k(r\cos\theta-h_n)]\frac{\cos kr}{(kr)^3} = -\frac{3k\cos kr\cos[\ \ ]}{(kr)^4}-\frac{k\sin kr\cos[\ \ ]}{(kr)^3}-\frac{k\cos\theta\cos kr\sin[\ \ ]}{(kr)^3}$$

$$\frac{\partial}{\partial r}\cos[k(r\cos\theta - h_n)]\frac{\cos kr}{(kr)^2}$$
$$= -\frac{2k\cos kr\cos[\ \ ]}{(kr)^3} - \frac{k\sin kr\cos[\ \ ]}{(kr)^2} - \frac{k\cos\theta\cos kr\sin[\ \ ]}{(kr)^2}$$

$$\frac{\partial}{\partial r}\cos[k(r\cos\theta - h_n)]\frac{\sin kr}{(kr)^2}$$
$$= -\frac{2k\sin kr\cos[\ \ ]}{(kr)^3} + \frac{k\cos kr\cos[\ \ ]}{(kr)^2} - \frac{k\cos\theta\sin kr\sin[\ \ ]}{(kr)^2}$$

$$\frac{\partial}{\partial r}\cos[k(r\cos\theta - h_n)]\frac{\cos kr}{kr}$$
$$= -\frac{k\cos kr\cos[\ \ ]}{(kr)^2} - \frac{k\sin kr\cos[\ \ ]}{kr} - \frac{k\cos\theta\cos kr\sin[\ \ ]}{kr}$$

$$\frac{\partial}{\partial r}\cos[k(r\cos\theta - h_n)]\frac{\sin kr}{kr}$$
$$= -\frac{k\sin kr\cos[\ \ ]}{(kr)^2} + \frac{k\cos kr\cos[\ \ ]}{kr} - \frac{k\cos\theta\sin kr\sin[\ \ ]}{kr}$$

$$\frac{\partial}{\partial r}\sin[k(r\cos\theta - h_n)]\frac{\cos kr}{(kr)^2}$$
$$= -\frac{2k\cos kr\sin[\ \ ]}{(kr)^3} - \frac{k\sin kr\sin[\ \ ]}{(kr)^2} + \frac{k\cos\theta\cos kr\cos[\ \ ]}{(kr)^2}$$

$$\frac{\partial}{\partial r}\sin[k(r\cos\theta - h_n)]\frac{\cos kr}{kr}$$
$$= -\frac{k\cos kr\sin[\ \ ]}{(kr)^2} - \frac{k\sin kr\sin[\ \ ]}{kr} + \frac{k\cos\theta\cos kr\cos[\ \ ]}{kr}$$

$$\frac{\partial}{\partial r}\sin[k(r\cos\theta - h_n)]\frac{\sin kr}{kr}$$
$$= -\frac{k\sin kr\sin[\ \ ]}{(kr)^2} + \frac{k\cos kr\sin[\ \ ]}{kr} + \frac{k\cos\theta\sin kr\cos[\ \ ]}{kr}$$

these can be used with Equation (3b) and Equation (10) of the main body to obtain the **full expression** for the radial force:

$$F_r = \pi E_0 k^3 a_p^3 a_s^3 \left\{ \frac{f_{1,p}}{2} \cos[k(r\cos\theta - h_n)] \left\{ f_{1,s} \cos kh_n \,(1 + 3\cos 2\theta) \left[\frac{3k\cos kr}{(kr)^4} + \frac{3k \sin kr}{(kr)^3} - \frac{k\cos kr}{(kr)^2} \right] + \frac{4}{3} f_{0,s} \sin kh_n \cos\theta \left[\frac{2k\cos kr}{(kr)^3} + \frac{2k\sin kr}{(kr)^2} - \frac{k\cos kr}{kr} \right] - f_{1,s}\cos kh_n\,(1+\cos 2\theta) \left[\frac{k\cos kr}{(kr)^2} + \frac{k \sin kr}{kr} \right] \right\} + \frac{2 f_{0,p}}{3} \cos[k(r\cos\theta - h_n)] \left\{ f_{1,s}\cos kh_n \cos\theta \left[-\frac{k\cos\theta\cos kr}{(kr)^2} - \frac{k\cos\theta \sin kr}{kr} \right] + \frac{2}{3} f_{0,s} \sin kh_n \left[-\frac{k\cos\theta\cos kr}{kr} \right] \right\} + \frac{f_{1,p}}{2} \sin[k(r\cos\theta - h_n)] \left\{ f_{1,s}\cos kh_n\,(1+3\cos 2\theta) \left[\frac{k\cos\theta\cos kr}{(kr)^3} + \frac{k\cos\theta\sin kr}{(kr)^2} \right] + \frac{4}{3} f_{0,s} \sin kh_n \cos\theta \left[\frac{k\cos\theta\cos kr}{(kr)^2} + \frac{k\cos\theta\sin kr}{kr} \right] - f_{1,s}\cos kh_n\,(1+\cos 2\theta) \left[\frac{k\cos\theta\cos kr}{kr} \right] \right\} + \frac{2 f_{0,p}}{3} \sin[k(r\cos\theta - h_n)] \left\{ f_{1,s}\cos kh_n \cos\theta \left[\frac{2k\cos kr}{(kr)^3} + \frac{2k\sin kr}{(kr)^2} - \frac{k\cos kr}{kr} \right] + \frac{2}{3} f_{0,s}\sin kh_n \left[\frac{k\cos kr}{(kr)^2} + \frac{k\sin kr}{kr} \right] \right\} \right\}$$

Expression of the force in the transversal plane can be obtained by substituting $\theta = \pi/2$ in the above. Also note that in this case

$$\cos\theta = 0$$

$$1 + 3\cos 2\theta = -2$$

$$1 + \cos 2\theta = 0$$

$$\cos[k(r\cos\theta - h_n)] = \cos kh_n$$

$$\sin[k(r\cos\theta - h_n)] = -\sin kh_n$$

and therefore

$$F_r = \pi E_0 k^3 a_p^3 a_s^3 \left\{ \frac{f_{1,p}}{2} \cos[\quad] \left\{ f_{1,s}\cos kh_n\,(1+3\cos 2\theta) \left[\frac{3k\cos kr}{(kr)^4} + \frac{3k\sin kr}{(kr)^3} - \frac{k\cos kr}{(kr)^2} \right] \right\} + \frac{2 f_{0,p}}{3} \sin[\quad] \left\{ \frac{2}{3} f_{0,s}\sin kh_n \left[\frac{k\cos kr}{(kr)^2} + \frac{k\sin kr}{kr} \right] \right\} \right\}$$
$$= \pi E_0 k^3 a_p^3 a_s^3 \left\{ f_{1,p} f_{1,s} \cos^2 kh_n \left[-\frac{3k\cos kr}{(kr)^4} - \frac{3k\sin kr}{(kr)^3} + \frac{k\cos kr}{(kr)^2} \right] - \frac{4}{9} f_{0,p} f_{0,s} \sin^2 kh_n \left[\frac{k\cos kr}{(kr)^2} + \frac{k\sin kr}{kr} \right] \right\}$$

which is equivalent to Equation (12) of the main text.

Similarly, for $\theta = 0$, i.e. along the z direction

$$\cos\theta = 1$$

$$1 + 3\cos 2\theta = 4$$

$$1 + \cos 2\theta = 2$$

and therefore

$$
\begin{aligned}
F_r = \pi E_0 k^3 a_p^3 a_s^3 \Bigg\{ & \frac{f_{1,p}}{2}\cos[\quad]\Bigg\{4 f_{1,s}\cos kh_n\left[\frac{3k\cos kr}{(kr)^4} + \frac{3k\sin kr}{(kr)^3} - \frac{k\cos kr}{(kr)^2}\right] \\
& + \frac{4}{3} f_{0,s}\sin kh_n\left[\frac{2k\cos kr}{(kr)^3} + \frac{2k\sin kr}{(kr)^2} - \frac{k\cos kr}{kr}\right] \\
& - 2 f_{1,s}\cos kh_n\left[\frac{k\cos kr}{(kr)^2} + \frac{k\sin kr}{kr}\right]\Bigg\} \\
& + \frac{2f_{0,p}}{3}\cos[\quad]\Bigg\{f_{1,s}\cos kh_n\left[-\frac{k\cos kr}{(kr)^2} - \frac{k\sin kr}{kr}\right] \\
& + \frac{2}{3} f_{0,s}\sin kh_n\left[-\frac{k\cos kr}{kr}\right]\Bigg\} \\
& + \frac{f_{1,p}}{2}\sin[\quad]\Bigg\{4 f_{1,s}\cos kh_n\left[\frac{k\cos kr}{(kr)^3} + \frac{k\sin kr}{(kr)^2}\right] \\
& + \frac{4}{3} f_{0,s}\sin kh_n\left[\frac{k\cos kr}{(kr)^2} + \frac{k\sin kr}{kr}\right] - 2 f_{1,s}\cos kh_n\left[\frac{k\cos kr}{kr}\right]\Bigg\} \\
& + \frac{2f_{0,p}}{3}\sin[\quad]\Bigg\{f_{1,s}\cos kh_n\left[\frac{2k\cos kr}{(kr)^3} + \frac{2k\sin kr}{(kr)^2} - \frac{k\cos kr}{kr}\right] \\
& + \frac{2}{3} f_{0,s}\sin kh_n\left[\frac{k\cos kr}{(kr)^2} + \frac{k\sin kr}{kr}\right]\Bigg\}\Bigg\}
\end{aligned}
$$

which simplifies to

$$
\begin{aligned}
F_r = \pi E_0 k^3 a_p^3 a_s^3 f_{1,s} \Bigg\{ & \frac{f_{1,p}}{2}\cos[kr]\Bigg\{4\left[\frac{3k\cos kr}{(kr)^4} + \frac{3k\sin kr}{(kr)^3} - \frac{k\cos kr}{(kr)^2}\right] \\
& - 2\left[\frac{k\cos kr}{(kr)^2} + \frac{k\sin kr}{kr}\right]\Bigg\} + \frac{2f_{0,p}}{3}\cos[kr]\Bigg\{\left[-\frac{k\cos kr}{(kr)^2} - \frac{k\sin kr}{kr}\right]\Bigg\} \\
& + \frac{f_{1,p}}{2}\sin[kr]\Bigg\{4\left[\frac{k\cos kr}{(kr)^3} + \frac{k\sin kr}{(kr)^2}\right] - 2\left[\frac{k\cos kr}{kr}\right]\Bigg\} \\
& + \frac{2f_{0,p}}{3}\sin[kr]\left[\frac{2k\cos kr}{(kr)^3} + \frac{2k\sin kr}{(kr)^2} - \frac{k\cos kr}{kr}\right]\Bigg\} \\
= \pi E_0 k^4 a_p^3 a_s^3 f_{1,s} \Bigg\{ & f_{1,p}\cos kr\left[\frac{6\cos kr}{(kr)^4} + \frac{6\sin kr}{(kr)^3} - \frac{3\cos kr}{(kr)^2} - \frac{\sin kr}{kr}\right] \\
& - \frac{2f_{0,p}}{3}\cos kr\left[\frac{\cos kr}{(kr)^2} + \frac{\sin kr}{kr}\right] \\
& + \left(f_{1,p} + \frac{2f_{0,p}}{3}\right)\sin kr\left[\frac{2\cos kr}{(kr)^3} + \frac{2\sin kr}{(kr)^2} - \frac{2\cos kr}{kr}\right]\Bigg\}
\end{aligned}
$$

when the nodes are aligned with the scatterer particle (h = 0). Note that this force is directly proportional to the dipole scattering coefficient of the scatterer particle.

Similarly, for the antinodal case ($h = \lambda/4$)

$$\cos[k(r - h_n)] = \cos\left[kr - \frac{\pi}{2}\right] = \sin kr$$

$$\sin[k(r - h_n)] = \sin\left[kr - \frac{\pi}{2}\right] = -\cos kr$$

Arriving at

$$\begin{aligned}
F_r = \pi E_0 k^3 a_p^3 a_s^3 f_{0,s} &\left\{\frac{f_{1,p}}{2}\sin[kr]\left\{\frac{4}{3}\left[\frac{2k\cos kr}{(kr)^3} + \frac{2k\sin kr}{(kr)^2} - \frac{k\cos kr}{kr}\right]\right\}\right. \\
&+ \frac{2f_{0,p}}{3}\sin[kr]\left\{\frac{2}{3}\left[-\frac{k\cos kr}{kr}\right]\right\} - \frac{f_{1,p}}{2}\cos[kr]\left\{\frac{4}{3}\left[\frac{k\cos kr}{(kr)^2} + \frac{k\sin kr}{kr}\right]\right\} \\
&\left. - \frac{2f_{0,p}}{3}\cos[kr]\left\{\frac{2}{3}\left[\frac{k\cos kr}{(kr)^2} + \frac{k\sin kr}{kr}\right]\right\}\right\} \\
&= \pi E_0 k^4 a_p^3 a_s^3 f_{0,s}\left\{\frac{2}{3}f_{1,p}\sin kr\left[\frac{2\cos kr}{(kr)^3} + \frac{2\sin kr}{(kr)^2}\right]\right. \\
&- \left(\frac{2}{3}f_{1,p} + \frac{4f_{0,p}}{9}\right)\sin kr\left[\frac{\cos kr}{kr}\right] \\
&\left. - \left(\frac{2}{3}f_{1,p} + \frac{4f_{0,p}}{9}\right)\cos kr\left[\frac{\cos kr}{(kr)^2} + \frac{\sin kr}{kr}\right]\right\}
\end{aligned}$$

Note that this force is directly proportional to the monopole scattering coefficient of the scatterer particle.

Secondary radiation force in the polar direction

The derivatives of the different terms containing θ:

$$\frac{\partial}{\partial\theta}\cos[k(r\cos\theta - h_n)]\,(1+3\cos 2\theta)$$
$$= -6\sin 2\theta\cos[\quad] + kr\sin\theta\,(1+3\cos 2\theta)\sin[\quad]$$

$$\frac{\partial}{\partial\theta}\cos[k(r\cos\theta - h_n)]\cos\theta = -\sin\theta\cos[\quad] + kr\sin\theta\cos\theta\sin[\quad]$$

$$\frac{\partial}{\partial\theta}\cos[k(r\cos\theta - h_n)]\,(1+\cos 2\theta) = -2\sin 2\theta\cos[\quad] + kr\sin\theta\,(1+\cos 2\theta)\sin[\quad]$$

$$\frac{\partial}{\partial\theta}\sin[k(r\cos\theta - h_n)]\cos\theta = -kr\sin\theta\cos\theta\cos[\quad] - \sin\theta\sin[\quad]$$

$$\frac{\partial}{\partial\theta}\sin[k(r\cos\theta - h_n)] = -kr\sin\theta\cos[\quad]$$

and on substitution to Equation (3b) and Equation (10):

$$\begin{aligned}
F_\theta = \frac{1}{r}\pi E_0 k^3 a_p^3 a_s^3 \Bigg\{ & \frac{f_{1,p}}{2}\cos[k(r\cos\theta - h_n)]\Bigg\{ f_{1,s}\cos kh_n \frac{\cos kr}{(kr)^3}[6\sin 2\theta] \\
& + \frac{4}{3}f_{0,s}\sin kh_n \frac{\cos kr}{(kr)^2}[\sin\theta] + f_{1,s}\cos kh_n\frac{\sin kr}{(kr)^2}[6\sin 2\theta] \\
& - f_{1,s}\cos kh_n\frac{\cos kr}{kr}[2\sin 2\theta] + \frac{4}{3}f_{0,s}\sin kh_n\frac{\sin kr}{kr}[\sin\theta]\Bigg\} \\
& + \frac{2f_{0,p}}{3}\cos[k(r\cos\theta - h_n)]\Bigg\{ f_{1,s}\cos kh_n\frac{\cos kr}{(kr)^2}[kr\sin\theta\cos\theta] \\
& + \frac{2}{3}f_{0,s}\sin kh_n\frac{\cos kr}{kr}[kr\sin\theta] + f_{1,s}\cos kh_n\frac{\sin kr}{kr}[kr\sin\theta\cos\theta]\Bigg\} \\
& + \frac{f_{1,p}}{2}\sin[k(r\cos\theta - h_n)]\Bigg\{ f_{1,s}\cos kh_n\frac{\cos kr}{(kr)^3}[-kr\sin\theta\,(1+3\cos 2\theta)] \\
& + \frac{4}{3}f_{0,s}\sin kh_n\frac{\cos kr}{(kr)^2}[-kr\sin\theta\cos\theta] \\
& + f_{1,s}\cos kh_n\frac{\sin kr}{(kr)^2}[-kr\sin\theta\,(1+3\cos 2\theta)] \\
& - f_{1,s}\cos kh_n\frac{\cos kr}{kr}[-kr\sin\theta\,(1+\cos 2\theta)] \\
& + \frac{4}{3}f_{0,s}\sin kh_n\frac{\sin kr}{kr}[-kr\sin\theta\cos\theta]\Bigg\} \\
& + \frac{2f_{0,p}}{3}\sin[k(r\cos\theta - h_n)]\Bigg\{ f_{1,s}\cos kh_n\frac{\cos kr}{(kr)^2}[\sin\theta] \\
& + f_{1,s}\cos kh_n\frac{\sin kr}{kr}[\sin\theta]\Bigg\}\Bigg\}
\end{aligned}$$

As all terms contain $\sin\theta$ or $\sin 2\theta$, the above force goes to zero when $\theta = 0$

However, when $\theta = \pi/2$, only terms $\cos\theta$, $\sin 2\theta$ or $1+\cos 2\theta$ disappear, leaving

$$
\begin{aligned}
F_\theta = \frac{1}{r}\pi E_0 k^3 a_p^3 a_s^3 \Bigg\{ & \frac{f_{1,p}}{2}\cos[\quad]\left\{\frac{4}{3} f_{0,s} \sin kh_n \frac{\cos kr}{(kr)^2}[\sin\theta]\right. \\
& \left. + \frac{4}{3} f_{0,s} \sin kh_n \frac{\sin kr}{kr}[\sin\theta]\right\} \\
& + \frac{2f_{0,p}}{3}\cos[\quad]\left\{\frac{2}{3} f_{0,s} \sin kh_n \frac{\cos kr}{kr}[kr\sin\theta]\right\} \\
& + \frac{f_{1,p}}{2}\sin[\quad]\left\{f_{1,s}\cos kh_n \frac{\cos kr}{(kr)^3}[-kr\sin\theta\,(1+3\cos 2\theta)]\right. \\
& \left. + f_{1,s}\cos kh_n \frac{\sin kr}{(kr)^2}[-kr\sin\theta\,(1+3\cos 2\theta)]\right\} \\
& + \frac{2f_{0,p}}{3}\sin[\quad]\left\{f_{1,s}\cos kh_n \frac{\cos kr}{(kr)^2}[\sin\theta] + f_{1,s}\cos kh_n \frac{\sin kr}{kr}[\sin\theta]\right\}\Bigg\}
\end{aligned}
$$

now evaluating the remaining terms, and simplifying:

$$\cos[k(r\cos\theta - h_n)] = \cos kh_n$$

$$\sin[k(r\cos\theta - h_n)] = -\sin kh_n$$

$$
\begin{aligned}
F_\theta = \frac{1}{r}\pi E_0 k^3 a_p^3 a_s^3 \sin 2kh_n \Bigg\{ & \frac{f_{1,p}}{3} f_{0,s}\left\{\frac{\cos kr}{(kr)^2} + \frac{\sin kr}{kr}\right\} + \frac{2f_{0,p}}{9} f_{0,s}\cos kr \\
& - \frac{f_{1,p}}{2} f_{1,s}\left\{\frac{\cos kr}{(kr)^2} + \frac{\sin kr}{kr}\right\} - \frac{f_{0,p}}{3} f_{1,s}\left\{\frac{\cos kr}{(kr)^2} + \frac{\sin kr}{kr}\right\}\Bigg\} \\
= \pi E_0 k^4 a_p^3 a_s^3 \frac{\sin 2kh_n}{18} \Bigg[& \left(6f_{1,p}f_{0,s} - 9f_{1,p}f_{1,s} - 6f_{0,p}f_{1,s}\right)\left(\frac{\cos kr}{(kr)^3} + \frac{\sin kr}{(kr)^2}\right) \\
& + 4f_{0,p}f_{0,s}\frac{\cos kr}{kr}\Bigg]
\end{aligned}
$$

which is only zero at either the nodes or antinodes, where $\sin 2kh_n = 0$.

Mesh convergence analysis

Mesh convergence analysis was carried out using a uniform and a non-uniform meshing to assess the convergence speed of the numerical method and verify its robustness. In both cases, we use a scaling parameter *mesh_size* to define the minimum and maximum discretization steps. For the uniform mesh, the maximum mesh size is given as $\lambda/\text{mesh\_size}$, while the minimum mesh size as $\lambda/(2 \cdot \text{mesh\_size})$. For the non-uniform mesh, the scattering and probe particles are meshed using the above minimum and maximum values, but the other domains (the fluid domain and the PML) are meshed using a coarser mesh, with minimum and maximum size of $\lambda/(0.6 \cdot \text{mesh\_size})$ and $\lambda/(1.2 \cdot \text{mesh\_size})$, respectively.

As the polystyrene particle in water has a lower contrast compared to the polystyrene particle in air, and consequently its relative secondary radiation force is lower, we chose this former case to analyze the mesh convergence. The results for various cases (node, antinode) and along different directions (radial and z) can be seen in the following Figs. S1-S4. The convergence error is defined as

$$\text{error} = \left| \frac{F_m - F_{m-1}}{F_m} \right|$$

where F_m denotes the secondary radiation force obtained using the mth mesh_size parameter. In all cases, the probe particle was placed 0.35λ distance from the scatterer, this distance is approximately halfway between the node and antinode and therefore has a non-zero force.

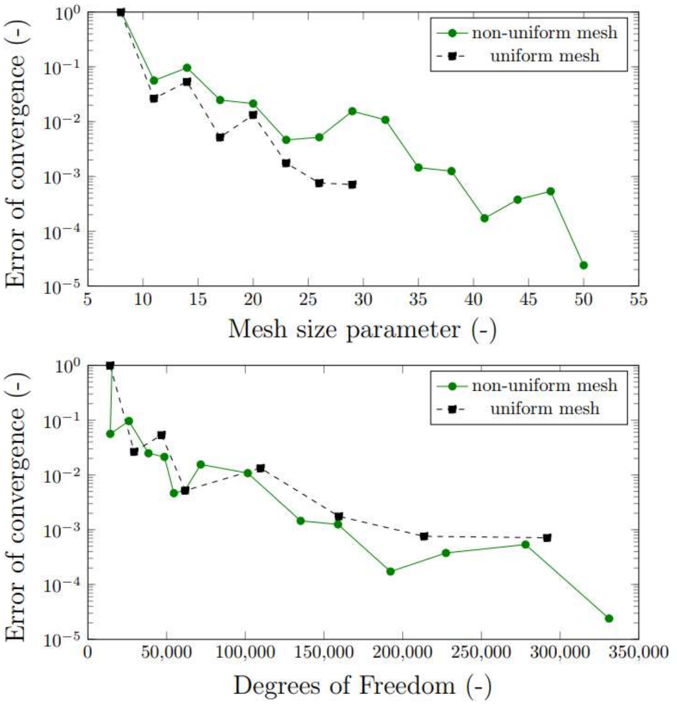

Figure S1. Convergence analysis results when the scattering particle is aligned with the pressure *antinode* and the probe particle is along the *z* direction. The convergence error is

plotted as a function of the mesh size parameter (top graph) and as a function of the number of degrees of freedom (bottom graph).

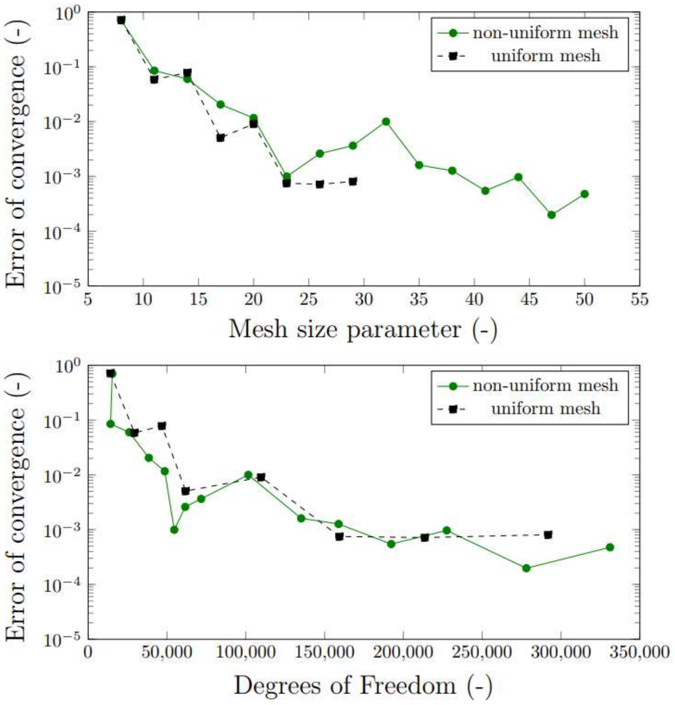

Figure S2. Convergence analysis results when the scattering particle is aligned with the pressure *node* and the probe particle is along the *z* direction. The convergence error is plotted as a function of the mesh size parameter (top graph) and as a function of the number of degrees of freedom (bottom graph).

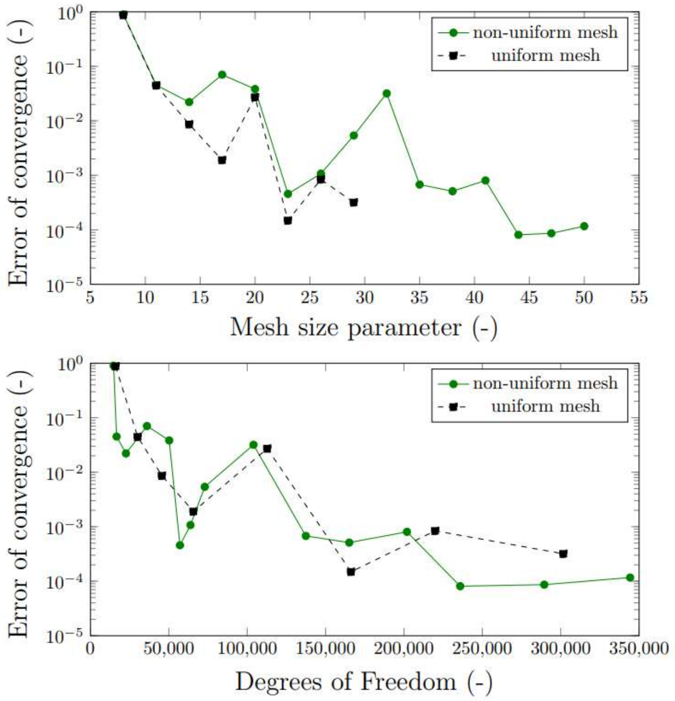

Figure S3. Convergence analysis results when the scattering particle is aligned with the pressure *antinode* and the probe particle is along the *r* direction. The convergence error is

plotted as a function of the mesh size parameter (top graph) and as a function of the number of degrees of freedom (bottom graph).

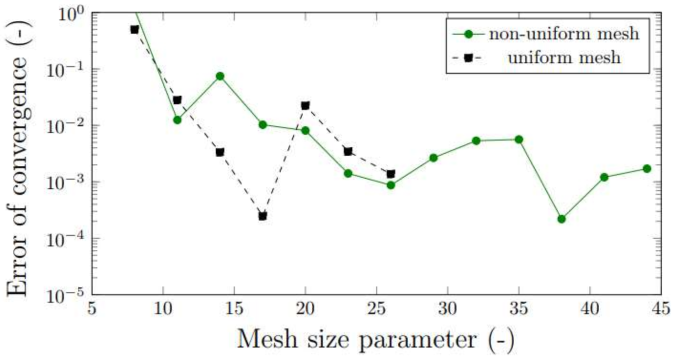

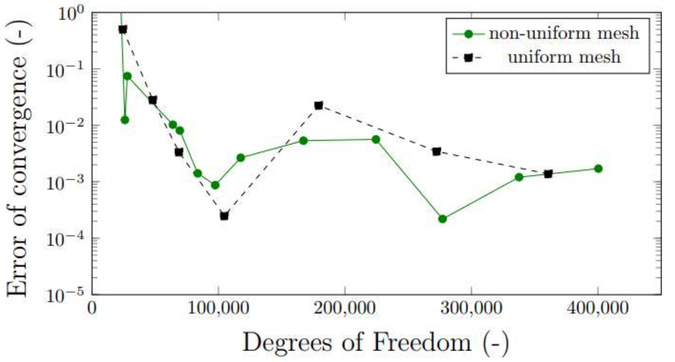

Figure S4. Convergence analysis results when the scattering particle is aligned with the pressure *node* and the probe particle is along the *r* direction. The convergence error is plotted as a function of the mesh size parameter (top graph) and as a function of the number of degrees of freedom (bottom graph).

Except for the last case (Figure S4), the convergence is fast, and no significant difference between the uniform and non-uniform mesh can be observed, as far as the number of degrees of freedom is concerned. For non-uniform mesh, for all four cases the error is below 1%, when mesh_size > 35, meaning that the particles are meshed between $0.0143\lambda < \text{mesh} < 0.0286\lambda$ and the other domains between $0.0238\lambda < \text{mesh} < 0.0476\lambda$.

For the uniform mesh, the error is below 1% for all cases when mesh_size > 23, meaning that for all domains the discretization size is $0.0217\lambda < \text{mesh} < 0.0435\lambda$.

As for the 2D case, increasing the number of degrees of freedom in the same order of magnitude as for the 3D case would require an inefficiently large mesh, we decided to use a quartic discretization in this case compared to the cubic discretization of the 3D case. The quartic discretization allows expansion of a medium-sized mesh into a large number of degrees of freedom using fourth order approximation of the solutions over each mesh element. Moreover, as for the 3D case no significant difference was observed between a uniform and non-uniform mesh, for the 2D investigations we only applied a simple uniform mesh with the same characteristics as before.

These results are summarized in Figures S5 and S6.

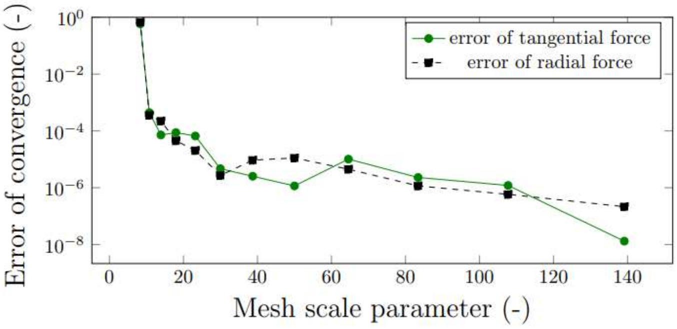

Figure S5. Convergence analysis results when the scattering particle is aligned with the pressure *node* and the probe particle is along either the r or z direction. The convergence error is plotted as a function of the mesh size parameter.

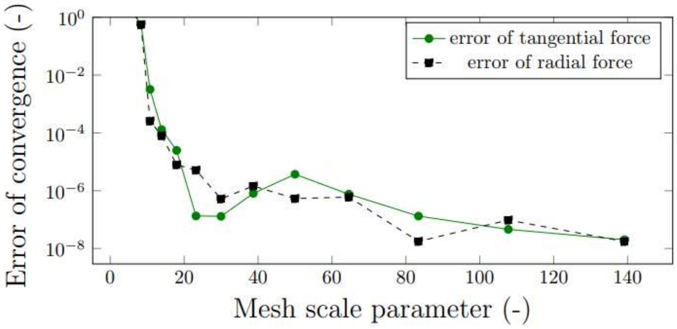

Figure S6. Convergence analysis results when the scattering particle is aligned with the pressure *antinode* and the probe particle is along either the r or z direction. The convergence error is plotted as a function of the mesh size parameter.

In both cases an extremely fast convergence can be observed irrespective of the particle position: the error is already less than 0.01% when the mesh scale parameter is 20. For the 2D case, the relationship between the mesh size parameter and the number of degrees of freedom is summarized in Table S1.

Table S1. Relation between number of degrees of freedom and mesh scale parameter for the 2D mesh convergence analysis

**mesh scale parameter**	**degrees of freedom**	**mesh scale parameter**	**degrees of freedom**
5	2304	29.97421	20714
6.457748	4070	38.71318	25338
8.340503	5166	50	38710
10.77217	6286	64.57748	60140
13.9128	11138	83.40503	95790
17.96907	15974	107.7217	156320
23.20794	18560	139.128	256354

Simulations for nearly touching spheres

Although the methods are not applicable for simulations of touching spheres, we show that the separation distance (the surface-to-surface distance) of the two spheres can be arbitrary low. In Figure S7, secondary radiation force results for PS particle in water, where the scatterer is at the antinode can be seen, showing separation distances as low as 0.001λ with successful simulation.

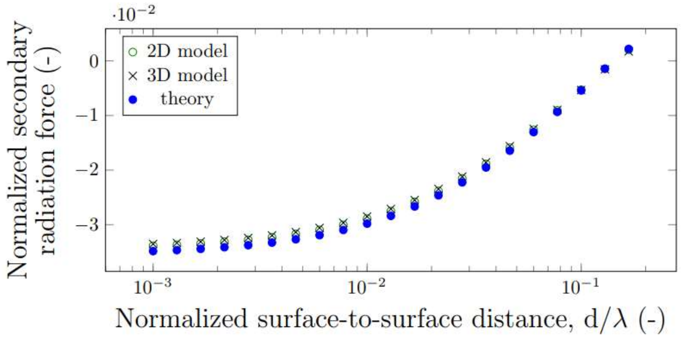

Figure S7. Secondary radiation force when the scattering particle is aligned with the pressure *antinode* and the probe particle is along the *z* direction. The distance in this case corresponds to surface-to-surface distance of the particles.
